# Histone demethylase JMJD2B/KDM4B regulates transcriptional program via distinctive epigenetic targets and protein interactors for the maintenance of trophoblast stem cells

**DOI:** 10.1038/s41598-020-79601-7

**Published:** 2021-01-13

**Authors:** Kylie Hin-Man Mak, Yuk Man Lam, Ray Kit Ng

**Affiliations:** School of Biomedical Sciences, Li Ka Shing Faculty of Medicine, The University of Hong Kong, Pokfulam, Hong Kong SAR China

**Keywords:** Stem cells, Differentiation, Developmental biology, Stem-cell differentiation

## Abstract

Trophoblast stem cell (TSC) is crucial to the formation of placenta in mammals. Histone demethylase JMJD2 (also known as KDM4) family proteins have been previously shown to support self-renewal and differentiation of stem cells. However, their roles in the context of the trophoblast lineage remain unclear. Here, we find that knockdown of *Jmjd2b* resulted in differentiation of TSCs, suggesting an indispensable role of JMJD2B/KDM4B in maintaining the stemness. Through the integration of transcriptome and ChIP-seq profiling data, we show that JMJD2B is associated with a loss of H3K36me3 in a subset of embryonic lineage genes which are marked by H3K9me3 for stable repression. By characterizing the JMJD2B binding motifs and other transcription factor binding datasets, we discover that JMJD2B forms a protein complex with AP-2 family transcription factor TFAP2C and histone demethylase LSD1. The JMJD2B–TFAP2C–LSD1 complex predominantly occupies active gene promoters, whereas the TFAP2C–LSD1 complex is located at putative enhancers, suggesting that these proteins mediate enhancer–promoter interaction for gene regulation. We conclude that JMJD2B is vital to the TSC transcriptional program and safeguards the trophoblast cell fate via distinctive protein interactors and epigenetic targets.

## Introduction

Placenta is an essential organ for fetal growth in mammals, providing an interface for the exchange of nutrients, oxygen, metabolites and other molecules between the fetal and maternal bloodstream. It is primarily formed by highly specialized cell types of the trophoblast lineage^1^. Abnormal development of trophoblast may result in inadequate placentation, which can lead to miscarriage, growth restriction, preeclampsia, other pregnancy disorders, and disease predisposition^2^. The trophoblast lineage is emerged from the first cell fate specification event in the embryo at the late morula stage, which results in the formation of trophectoderm (TE) and inner cell mass (ICM). TE cells contribute to the formation of the placenta, whereas ICM cells give rise to the embryo proper. Cells committed to the embryonic lineages are strictly excluded from the trophoblast fate, and vice versa^3–5^. The restricted lineage specification is recapitulated in the in vitro derived embryonic stem cell (ESC) and trophoblast stem cell (TSC) from ICM and TE, respectively. TSC serves as an excellent in vitro stem cell model for the study of trophoblast development. The multipotent and self-renewal properties of TSC are associated with the trophoblast-specific transcriptional program, including the expression of key transcription factors CDX2, EOMES, ELF5, TFAP2C, ETS2, SOX2, and ESRRB. It has been shown that many of these transcription factors interact with each other, for example, TFAP2C interacts with SOX2 and ELF5^6,7^, to reinforce their expression through a positive feedback loop and to maintain the stem cell circuit by cross-regulation^8–11^.

Lineage specification and the associated gene expression pattern during cell differentiation are tightly associated with epigenetic regulations. It is noted that the global DNA methylation level in TE cells is relatively lower than that of ICM cells^12^. DNA hypomethylation can facilitate cell fate switch from embryonic to trophoblast lineage, as evidenced by the presence of trophoblast marker expressing cells in *Dnmt1*^−/−^ embryos and in DNA methylation-deficient ESCs^11^. The differential DNA methylation of transcription factor *Elf5* was proposed to be responsible for safeguarding the commitment of trophoblast cell fate in TSCs. However, the restricted expression of other key trophoblast stemness genes, such as *Cdx2* and *Eomes*, was not regulated by DNA methylation in both ESCs and TSCs^11^, suggesting the involvement of other epigenetic mechanisms in trophoblast lineage specification and maintenance. Importantly, repressive histone modification H3K9me3 has been shown to have a crucial role in the transcriptional regulation of trophoblast lineage genes, which contributes to the trophoblast/embryonic lineage barrier in ESCs. It has been shown that genetic abrogation of histone methyltransferase *Setdb1* (also known as *Eset* or *Kmt1e*) in ESCs resulted in *trans*-differentiation to TSC-like cells, which reveals the importance of H3K9me3 for the repression of the trophoblast transcriptional program in ESCs^13,14^. In addition, a negative correlation between promoter H3K9me3 and gene expression of several key stemness factors was observed during TSC differentiation^15^, further supporting the epigenetic regulatory role of H3K9me3 in trophoblast development. However, little is known about the global epigenetic alterations in association with the transcriptional program in the maintenance of TSCs.

JMJD2 (also known as KDM4) family belongs to a group of Jmj-domain-containing proteins, which is evolutionarily conserved from budding yeast to mammals^16,17^. All four members of the JMJD2 family have the ability to demethylate H3K9me2/3 and/or H3K36me2/3. It has been reported that JMJD2 family members are involved in the regulation of stem cell self-renewal and differentiation. JMJD2C maintains the expression of pluripotent gene *Nanog* in ESCs by preventing the deposition of repressive H3K9 methylation^18^. A recent genome-wide study showed that JMJD2A and JMJD2C act together at active gene promoters for the maintenance of the transcriptional program in ESCs^19^. JMJD2A was also reported to be essential for the derepression of *Myog* during skeletal muscle cell differentiation^20^, whereas JMJD2B was involved in the osteogenic differentiation of mesenchymal stem cell through mediating H3K9me3 demethylation at the *DLX* promoter^21^. Importantly, several studies using ESC models demonstrated the binding of JMJD2 proteins at trophoblast lineage gene loci, such as *Cdx2*, *Eomes*, *Tfap2c*, *Hand1*, *Ovol2*, and *Gcm1*^19,22,23^, suggesting a repressive role of JMJD2 to the trophoblast cell fate in ESCs. Whether JMJD2 family is epigenetically involved in the maintenance of stemness in TSCs remains to be elucidated.

In this study, we show that JMJD2B is a key JMJD2 family member regulating the TSC transcriptional program. JMJD2B is persistently bound at the majority of active gene loci during TSC differentiation, whereas H3K9me3 is mainly associated with non-trophoblast lineage genes and keeps them silent. Unlike in ESCs, we do not observe any global negative correlation between JMJD2B and H3K9me3 in TSCs. In a subset of silent genes, JMJD2B is co-enriched with H3K9me3 and is associated with the loss of H3K36me3 mark. By characterizing the JMJD2B binding motifs and other transcription factor binding profiles, we discover a novel interaction between JMJD2B, AP-2 family transcription factor TFAP2C, and histone demethylase LSD1 at active gene promoters. Taken together, our data reveal that JMJD2B regulates the transcriptional program via distinctive interacting partners and epigenetic mechanisms that are crucial to the lineage identity of TSCs.

## Results

### JMJD2 family is critical for the maintenance of undifferentiated TSC state

**Figure 1:**
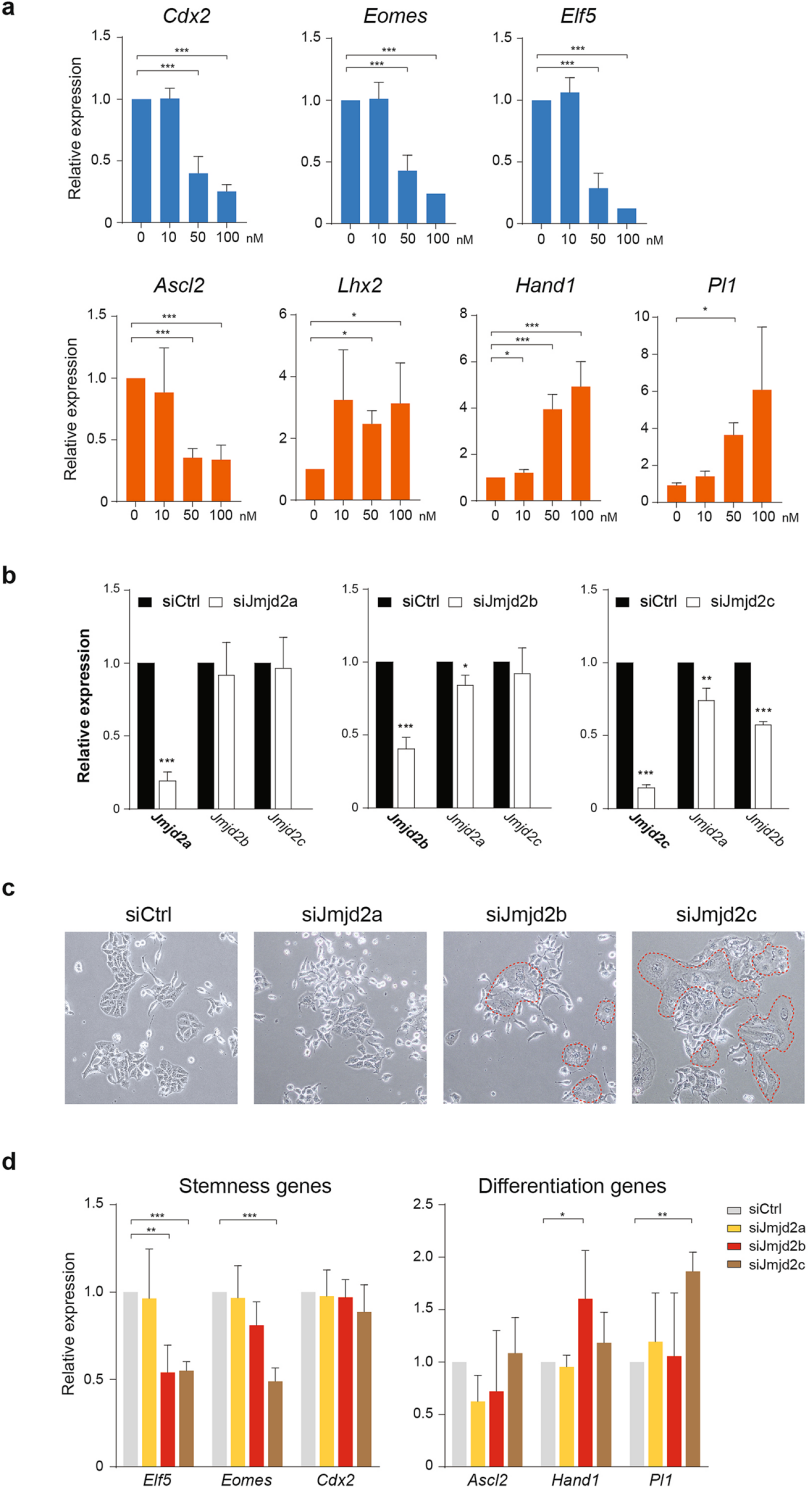
Inhibition of JMJD2 proteins induces TSC differentiation. (**a**) TSCs treated with pan-selective inhibitor of Jumonji histone demethylases JIB-04 exhibit downregulation of stem cell markers (*Cdx2*, *Eomes* and *Elf5*) and early differentiation marker *Ascl2*, and upregulation of differentiation markers (*Lhx2*, *Hand1* and *Pl1*). (**b**) *Jmjd2a*, *Jmjd2b*, or *Jmjd2c* was knockdown in TSCs by siRNA. Reduced gene expression at the mRNA level was confirmed by qRT-PCR analysis. (**c**) Cytoplasmic and nuclear-enlarged giant cell-like cells (encircled) were observed in *Jmjd2b*- or *Jmjd2c*-knockdown TSCs. (**d**) Knockdown of *Jmjd2b* significantly downregulated stem cell gene *Elf5* and upregulates differentiation gene *Hand1* expression, while knockdown of *Jmjd2c* significantly downregulated both *Elf5* and *Eomes*, and upregulate differentiation factor *Pl1* expression. Three independent experiments were performed with biological replicates of each sample. Data represent mean ± SD; Student’s t-test *P < 0.05, **P < 0.01, and ***P < 0.001.

Previous studies of murine ESCs showed that manipulation of H3K9 methyltransferase can induce trophoblast lineage fate^13,14^. To ask whether the JMJD2 family is essential in TSCs, we treated TSCs with JIB-04, which is a non-competitive inhibitor of α-ketoglutarate and serves as a pan-selective inhibitor of Jumonji histone demethylases, including KDM5/JARID1 family, KDM4/JMJD2 family, and KDM6B/JMJD3^24–26^. Although the IC_50_ values of JIB-04 ranged from 230 to 1100 nM for different histone demethylases^24^, we observed that TSCs exhibited growth inhibition and underwent apoptosis at 250 nM and 500 nM concentrations, respectively (data not shown). Therefore, we attempted to treat TSCs at a concentration ranged from 0–100 nM, which was reported to show biological responses on cell proliferation and gene expression^26–28^. Interestingly, it was observed that stemness-associated genes, including *Cdx2*, *Eomes*, and *Elf5*, were significantly downregulated by 2.5–3.5 folds at 50 nM JIB-04 treatment for 48 h, and their expressions were further decreased at 100 nM treatment in a dosage-dependent manner (Fig. 1a). A similar reduction pattern was also observed for the early differentiation marker, *Ascl2*, as opposed to the significant induction of late differentiation markers, *Lhx2*, *Hand1*, and *Pl1* (Fig. 1a). These results suggest that inhibition of Jmonji histone demethylases by JIB-04 treatment promotes differentiation of TSCs.

To examine the role of individual JMJD2 family members in the maintenance of TSC, we transfected TSCs with siRNA and examined the transient knockdown efficiency using quantitative RT-PCR after 72 h (Fig. 1b). Strikingly, a relatively higher portion of *Jmjd2b*- or *Jmjd2c*-depleted TSCs showed a flattened and enlarged differentiated cell morphology of trophoblast giant cells (TGCs), whereas *Jmjd2a*-depleted TSCs showed no morphological changes (Fig. 1c). Consistent with the observed cell morphological changes, the expression level of stem cell factor *Elf5* was significantly reduced in both *Jmjd2b*-knockdown and *Jmjd2c*-knockdown cells, as compared with the siRNA control sample. Another stem cell gene *Eomes* was also downregulated in the *Jmjd2c-*knockdown sample (Fig. 1d). Interestingly, the expression levels of differentiation markers, *Hand1* and *Pl1*, were found to be elevated in *Jmjd2b-*knockdown and *Jmjd2c-*knockdown cells, respectively (Fig. 1d). In contrast to the knockdown of *Jmjd2b* or *Jmjd2c*, the gene expression levels of all marker genes in the *Jmjd2a-*knockdown cells were comparable to the siRNA control. As the siRNA for *Jmjd2c* also exhibited a mild reduction of *Jmjd2a* and *Jmjd2b* expression (Fig. 1b), JMJD2B was selected in our study to further examine its cellular functions in TSCs.

**Figure 2:**
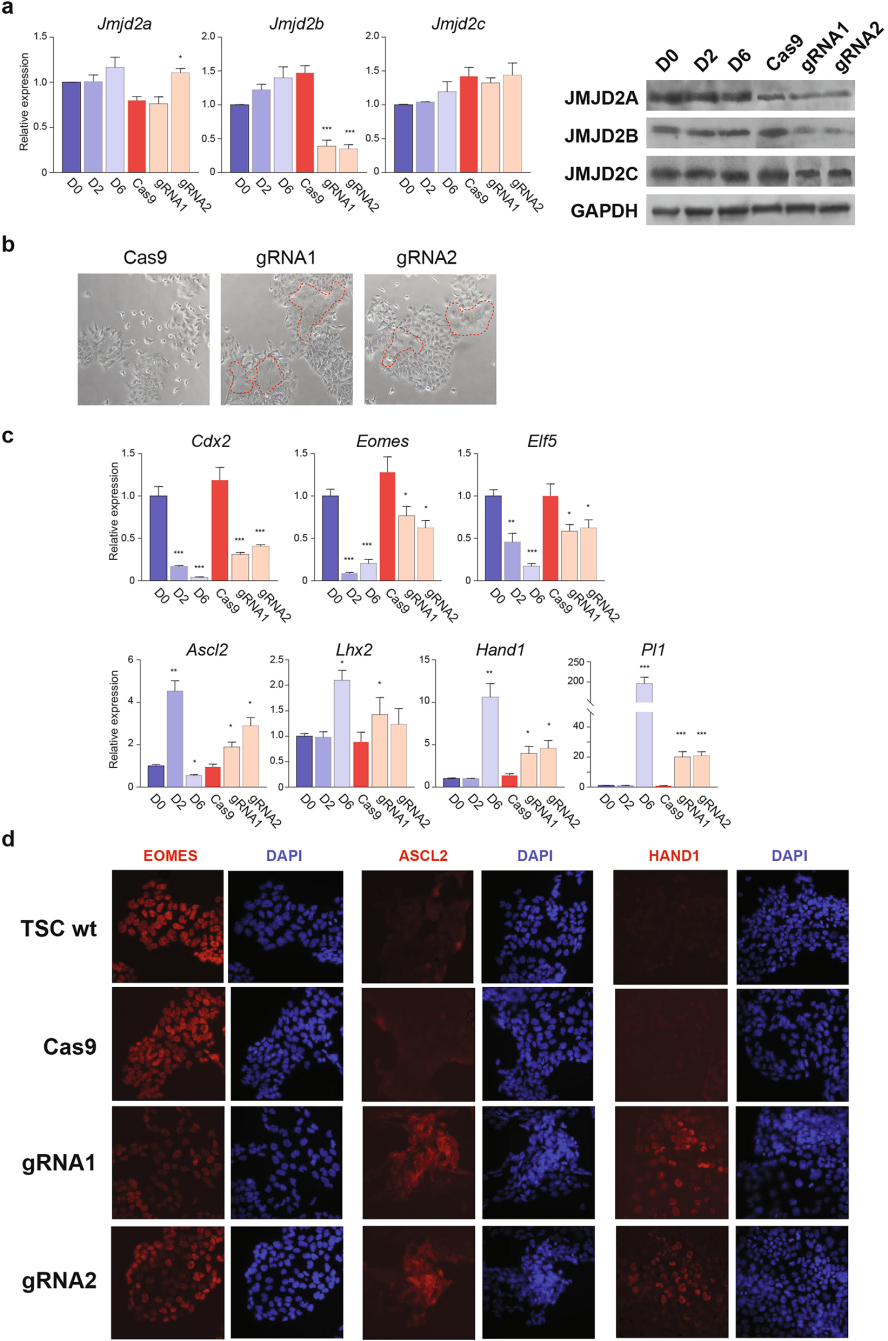
Depletion of JMJD2B prone TSCs to loss stemness. (**a**) qRT-PCR (left) and Western blotting (right) show stable expression of JMJD2 during TSC differentiation. Depletion of JMJD2B is observed in two CRISPR-Cas9 genome edited TSC lines (gRNA1 and 2), when compared to the control line (Cas9) (without gRNA), without affecting the expression of JMJD2A and JMJD2C. The un-processed full-length images of Western blot result are not available for the cropped blots shown. (**b**) CRISPR knockdown of *Jmjd2b* shows higher portion of cells with differentiated cell morphology (encircled). (**c**) mRNA expression of stemness makers and differentiation markers in the CRISPR knockdown cells. The degree of expression changes is less than that from the in vitro differentiated TSCs. Three independent experiments were performed with biological replicates of each sample. Data represent mean ± SD; Student’s t-test *P < 0.05, **P < 0.01, and ***P < 0.001. (**d**) Immunostaining patterns of undifferentiated (EOMES), early (ASCL2) and late (HAND1) differentiation markers. A subset of CRISPR knockdown cells show alteration of marker protein expression, suggesting they are differentiating. Photographs were taken at ×400 magnification and the same exposure time applies to all sample for each marker. D0, undifferentiated; D2, 2-day differentiated; D6, 6-day differentiated TSCs.

We notice that the transient knockdown of *Jmjd2b* by siRNA triggered only a mild induction of *Hand1* and no alterations of *Cdx2* expression. Therefore, we established two stable *Jmjd2b-*knockdown TSC lines using the CRISPR-Cas9 genome editing approach. Two guide RNA (gRNA) sequences were designed to target the exon 3 and 5 of the *Jmjd2b* gene, which could introduce frameshift mutations or truncation at the JmjC domain. The transfection condition of gRNA was optimized to achieve over 95% transfection efficiency, which presumably resulted in genome editing at the *Jmjd2b* locus in the majority of TSCs. Both CRISPR knockdown TSC lines showed depletion of the JMJD2B without altering the expression of JMJD2A and JMJD2C (Fig. 2a). The CRISPR knockdown lines showed a higher portion of cells with TGC morphology (Fig. 2b). Importantly, we observed a consistent reduction of stemness markers (*Cdx2*, *Eomes*, and *Elf5*) and induction of differentiation markers (*Ascl2*, *Lhx2*, *Hand1*, and *Pl1*) in the CRISPR knockdown cells, even though the mRNA levels of these markers were not as strong as the in vitro differentiated TSCs (as compared to the day 2 or day 6 in vitro differentiated samples) (Fig. 2c). The immunostaining patterns of marker proteins further demonstrated that only a subset of CRISPR knockdown cells potentially underwent differentiation (Fig. 2d). Together, these results suggest that depletion of JMJD2B may prone TSCs to exit the stem cell state and undergo differentiation.

### Jmjd2b binding, but not H3K9me3 enrichment, is associated with TSC biology

To investigate the epigenetic regulation mediated by JMJD2B in TSC maintenance, we profiled the genome-wide JMJD2B occupancy and its association with H3K9me3 in TSCs by JMJD2B- and H3K9me3-ChIP followed by high-throughput sequencing (ChIP-seq). Globally, we identified 272,434, 225,041, and 304,499 JMJD2B binding sites in the undifferentiated (D0), 2-day differentiated (D2), and 6-day differentiated (D6) TSCs, respectively. The general overview of JMJD2B-mediated functions was revealed by Genomic Regions Enrichment of Annotations Tool (GREAT) analysis using the top 65,000 confidence peaks in each TSC sample (Supplementary Fig. 1 and Supplementary Table 1). In D0 TSC, JMJD2B was bound to the loci associated with blastocyst formation, stem cell maintenance, and TGF-β pathway, whereas during differentiation, its binding shifted to the loci associated with heterochromatin organization, cell cycle, and pathways involved in placental development, such as PI3K, FAS, and EGF receptor signaling^29–32^. The biological term “trophectodermal cell differentiation” was also strongly associated with the D0 (undifferentiated) and D6 (most differentiated) samples. Notably, several inactive pathways, such as Notch signaling, gonadotropin-releasing hormone receptor pathway, and integrin signaling, were enriched, suggesting that JMJD2B could have a dual function by activating stemness factors while suppressing pro-differentiation signals at the undifferentiated state. In addition, similar Gene Ontology (GO) terms were observed between D2 and D6 samples, which were distinct from the D0 sample. The strong dynamic changes of GO annotation at the early differentiation process (D0 vs. D2/6) implies that JMJD2B could have a unique regulatory role in the undifferentiated TSCs. Once TSCs undergo differentiation, the functions of JMJD2B could remain stable.

For H3K9me3, we identified 254,126, 217,900, and 244,477 enriched regions in D0, D2 and D6 TSCs, respectively. Functional annotation of the H3K9me3 regions in TSCs demonstrated no significant enrichment of the terms associated with the trophoblast lineage biology, regardless of the differentiation status (Supplementary Fig. 1 and Supplementary Table 1). Many of the loci enriched with H3K9me3 in TSCs for all of the three time points were indeed associated with metabolic processes, such as phospholipase activity, adenylate cyclase activity, and glutamate receptor pathway. As a result, the highly similar GO annotation from D0 to D6 suggests that H3K9me3 is not dynamically regulated during TSC differentiation. The apparent differences of the GO annotations between JMJD2B and H3K9me3 ChIP-seq data also suggest that they are not targeting the same set of genes in TSCs. Importantly, the global correlation analysis of the ChIP-seq signals at gene promoters further demonstrated that JMJD2B and H3K9me3 were only weakly correlated to each other in TSCs (Supplementary Fig. 2).

### H3K9me3 regulates a small number of key TSC factors

Since the H3K9me3-enriched loci were not associated with biological processes that are relevant to the trophoblast lineage, we attempted to reveal the global correlation between the H3K9me3 enrichment and transcriptional repression in TSCs. The mRNA expression profiles of the corresponding TSC samples were generated by RNA-seq. It was observed that 2,486 upregulated genes and 2,154 downregulated genes in the early differentiation phase (from D0 to D2), and 3,261 upregulated genes and 2,754 downregulated genes in the late differentiation phase (from D2 to D6) (Supplementary Fig. 3 and Supplementary Table 2). The expression profiles showed a sharp decrease in the expression of trophoblast stem cell factors (*Cdx2*, *Eomes*, *Elf5*, *Esrrb*, and *Sox2*), followed by an increase in the expression of early differentiation markers (*Ascl2*, *Ovol2*, and *Tfeb*) at D2 TSC, and late differentiation markers for TGCs (*Pl1*, *Pl2*, *Prl8a9*, and *Ctsq*) and syncyiotrophoblast (*Syna*) at D6 TSC, respectively.

**Figure 3:**
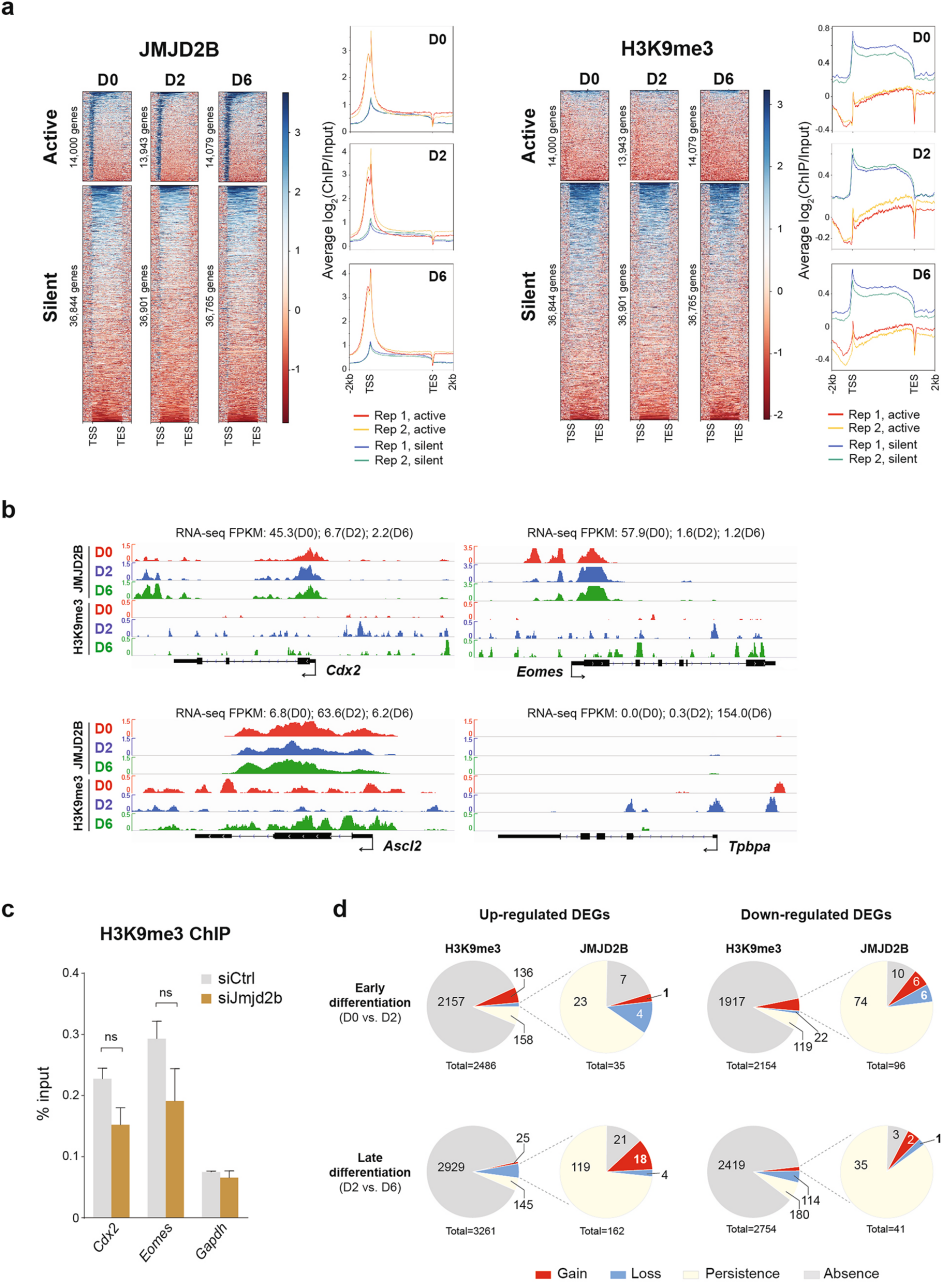
Global correlation between JMDJ2B and H3K9me3 during TSC differentiation. (**a**) Heatmaps showing JMJD2B (left) and H3K9me3 (right) enrichment level at transcriptionally active or silent gene loci in differentiating TSC samples. The average ChIP-seq signal patterns of active and silent genes were shown next to the corresponding heatmaps. zFPKM value (from RNA-seq) over -3 was regarded as transcriptionally active genes. Genes include both protein coding and non-coding from the *Mus musculus* mm10 (Ensembl release 89). *TSS* transcriptional start site, *TES* transcriptional end site. (**b**) ChIP-seq signal profiles at TSC factors, *Cdx2* and *Eomes*, and differentiation factors, *Ascl2* and *Tpbpa*. RNA-seq FPKM values are shown to indicate negative correlation between expression and H3K9me3 patterns. (**c**) ChIP-qPCR showing no significant changes of H3K9me3 enrichment at *Cdx2* and *Eomes* promoters in the *Jmjd2b* knockdown TSCs when compared to that of the control sample. *Gapdh* promoter served as negative enrichment control. Experiments were performed with three replicates. Data represent mean ± SD; ns: not significant. (**d**) Pie charts showing the global patterns of H3K9me3 enrichment at DEGs. Only small subsets of DEGs that show negative correlation with H3K9me3 have reverse pattern of JMJD2B binding. “Gain” is defined by “from absence to presence”; “loss” is defined by “from presence to absence”.

By defining the transcriptional status using a normalization matrix developed by Hart et al*.*^33^, there were around 14,000 transcriptionally active genes and around 36,800 transcriptionally silent genes (including protein coding and non-coding) in each of the three stages of TSCs. Strikingly, JMJD2B was enriched at the promoters of the vast majority of actively transcribing genes, as opposed to the H3K9me3 pattern (Fig. 3a and Supplementary Fig. 4a). It is noted that genes with significantly higher transcript per million mapped reads (FPKM) values were enriched with JMJD2B but not H3K9me3, in comparison to those with JMJD2B and H3K9me3 co-enrichment or H3K9me3 enrichment per se at their promoters (Supplementary Fig. 4b). Although H3K9me3 is known as a repressive epigenetic mark, only two TSC factors (*Cdx2* and *Eomes*) and two differentiation factors (*Ascl2* and *Tpbpa*) in a panel of 17 selected trophoblast lineage genes showed a negative correlation between H3K9me3 and gene expression during TSC differentiation (Fig. 3b). The expression of the rest of 13 trophoblast lineage genes (*Elf5*, *Esrrb*, *Sox2*, *Dlx3*, *Ovol2*, *Cdkn1c*, *Tfeb*, *Syna*, *Ctsq*, *Pl1*, *Pl2*, *Tpbpb*, *Prl8a9*) showed no correlation with the H3K9me3 pattern. We also observed that genes with persistence or absence of H3K9me3 during TSC differentiation showed a wider range of transcriptional output (including up- and down-regulation) when compared to genes with differential H3K9me3 enrichment (Supplementary Fig. 4c). A similar observation of transcriptional output patterns can also be found at the JMJD2B bound genes (Supplementary Fig. 4d). Interestingly, among the four genes (*Cdx2*, *Eomes*, *Ascl2*, and *Tpbpa*) which showed negative correlations between gene expression and H3K9me3, their JMJD2B patterns remained unchanged (Fig. 3b). We further demonstrated that the H3K9me3 enrichment remained at a low level at the *Cdx2* and *Eomes* loci in the *Jmjd2b-*knockdown TSCs (Fig. 3c), indicating that the H3K9me3 pattern is independent of JMJD2B binding. Globally, the gene expression of about 3% (334/10,655) differentially expressed genes (DEGs) was associated negatively with the repressive H3K9me3 mark during TSC differentiation. However, only 26 of these genes demonstrated a reverse correlation between JMJD2B and H3K9me3 patterns, while the majority of them showed persistent JMJD2B binding (Fig. 3d). Together, these data indicate that there is a lack of functional correlation between JMJD2B and H3K9me3 in TSC gene regulation.

### JMJD2B cooperates with H3K9me3 for stable silencing of embryonic lineage genes

**Figure 4:**
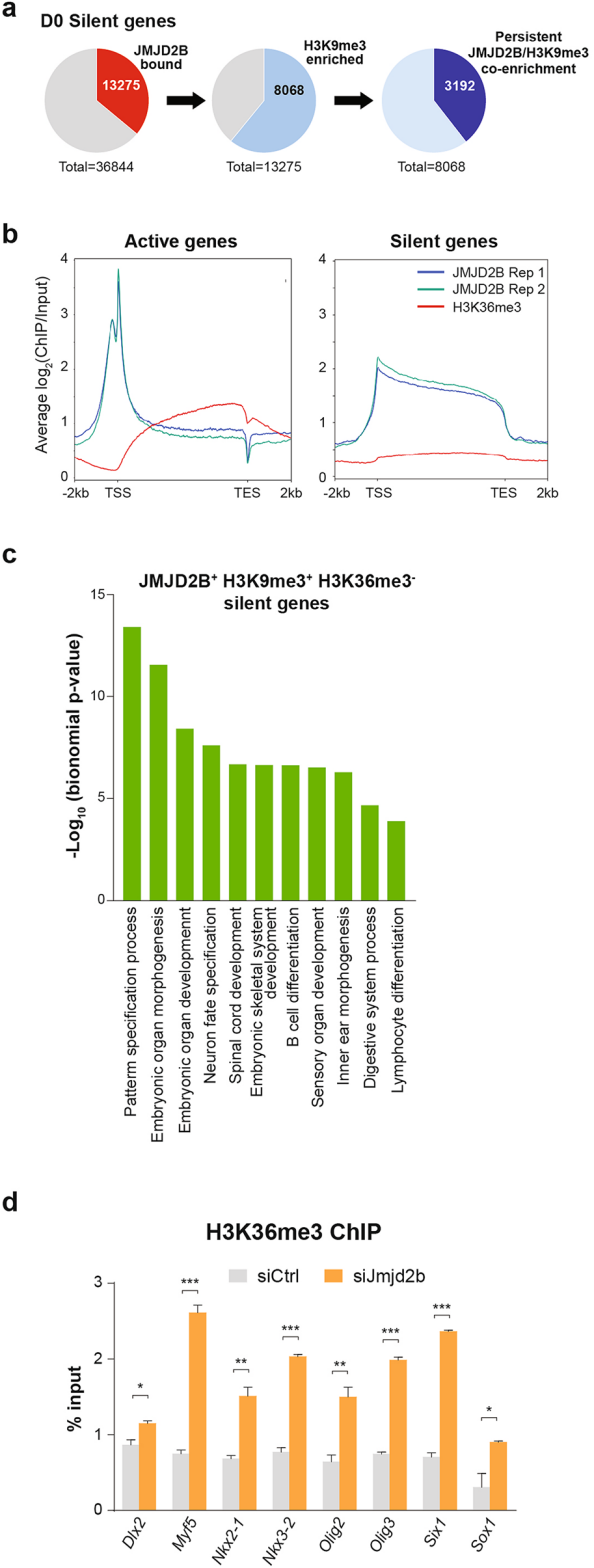
JMJD2B represses non-trophoblast genes through H3K36me3 demethylation. (**a**) Pie charts showing co-enrichment of JMJD2B and H3K9me3 in a subset of silent genes (8,068) in undifferentiated TSCs. 3,192 of these co-enriched genes show persistent patterns throughout TSC differentiation (from D0 to D6). (**b**) The average ChIP-seq signal patterns of JMJD2B and H3K36me3 in undifferentiated TSCs. *TSS* transcriptional start site, *TES* transcriptional end site. (**c**) GO analysis of the biological processes for the JMJD2B^+^ H3K9me3^+^ H3K36me3^-^ silent genes in undifferentiated TSCs. (**d**) ChIP-qPCR showing significant increase in gene body H3K36me3 enrichment at a panel of H3K9me3-associated embryonic lineage genes in the *Jmjd2b* knockdown TSCs when compared to that of the control sample. The qPCR primers were designed at the gene bodies based on H3K36me3 ChIP-seq signal. Three independent experiments were performed. Data represent mean ± SD; Student’s t-test *P < 0.05, **P < 0.01, and ***P < 0.001.

For the 36% (13,275) of the silent genes bound by JMJD2B in the undifferentiated TSCs, it is noticed that the majority of them (61%, 8,068) were also marked by H3K9me3. Unexpectedly, about 40% (3,192) of these genes remained JMJD2B/H3K9me3 co-enriched and silent throughout the TSC differentiation process (Fig. 4a). The co-enrichment of JMJD2B and H3K9me3 at these stable silent genes indicates that the epigenetic function of JMJD2B in gene repression is not mediated through modulation of H3K9me3. Since H3K36me3 is another histone target of the JMJD2 family, we re-analyzed a published H3K36me3 ChIP-seq dataset from TSCs^34^ and compared it to our in-house JMJD2B pattern. The enrichment of H3K36me3 mark at gene body is significantly associated with genes that have higher FPKM values (Supplementary Fig. 5a). It is interesting to note that while JMJD2B was mainly enriched at the promoters of transcriptionally active genes, their gene bodies had low JMJD2B enrichment but with a strong H3K36me3 level. A reverse pattern of JMJD2B and H3K36me3 was also observed at the gene bodies of a subset of transcriptionally silent genes, where JMJD2B was enriched in the absence of the H3K36me3 mark (Fig. 4b and Supplementary Fig. 5b). Together with the H3K9me3 binding patterns, genes with promoter JMJD2B and gene body H3K36me3 marks, but without promoter H3K9me3 (JMJD2B^+^ H3K9me3^-^ H3K36me3^+^), have higher FPKM values in TSCs (Supplement Fig. 5c). Importantly, the silent genes with JMJD2B and H3K9me3 co-enrichment, but without H3K36me3 (JMJD2B^+^ H3K9me3^+^ H3K36me3^−^), did not contain any prominent trophoblast lineage genes. GO analysis showed that this subset of genes was strongly associated with a wide range of embryonic developmental processes (Fig. 4c). On the contrary, JMJD2B-associated silent genes without both H3K9me3 and H3K36me3 (JMJD2B^+^ H3K9me3^−^ H3K36me3^−^) were mainly involved in immune response and metabolic processes (Supplementary Fig. 5d). To examine the correlation between JMJD2B binding and the H3K36me3 pattern, we selected a panel of silent embryonic lineage genes that showed persistent JMJD2B binding during TSC differentiation (from D0 to D6). It is noted that knockdown of *Jmjd2b* in TSCs resulted in an elevation of H3K36me3 level at the gene bodies in this panel of embryonic lineage genes (Fig. 4d), which supports the function of JMJD2B is negatively associated with the H3K36me3 level. Although *Jmjd2b* knockdown in TSCs resulted in a twofold increased H3K36me3 level, all these embryonic genes showed no alterations in gene expression (Supplementary Fig. 5e). It thus suggests that the presence of other embryonic lineage transcription factors is required for their transcriptional activation, or the presence of other repressive factors persists which keeps them silent. Collectively, these results show that JMJD2B modulates the H3K36me3 level at those H3K9me3-associated non-trophoblast genes to safeguard the lineage fate of TSCs.

### JMJD2B interacts with the TFAP2C–LSD1 complex

**Figure 5:**
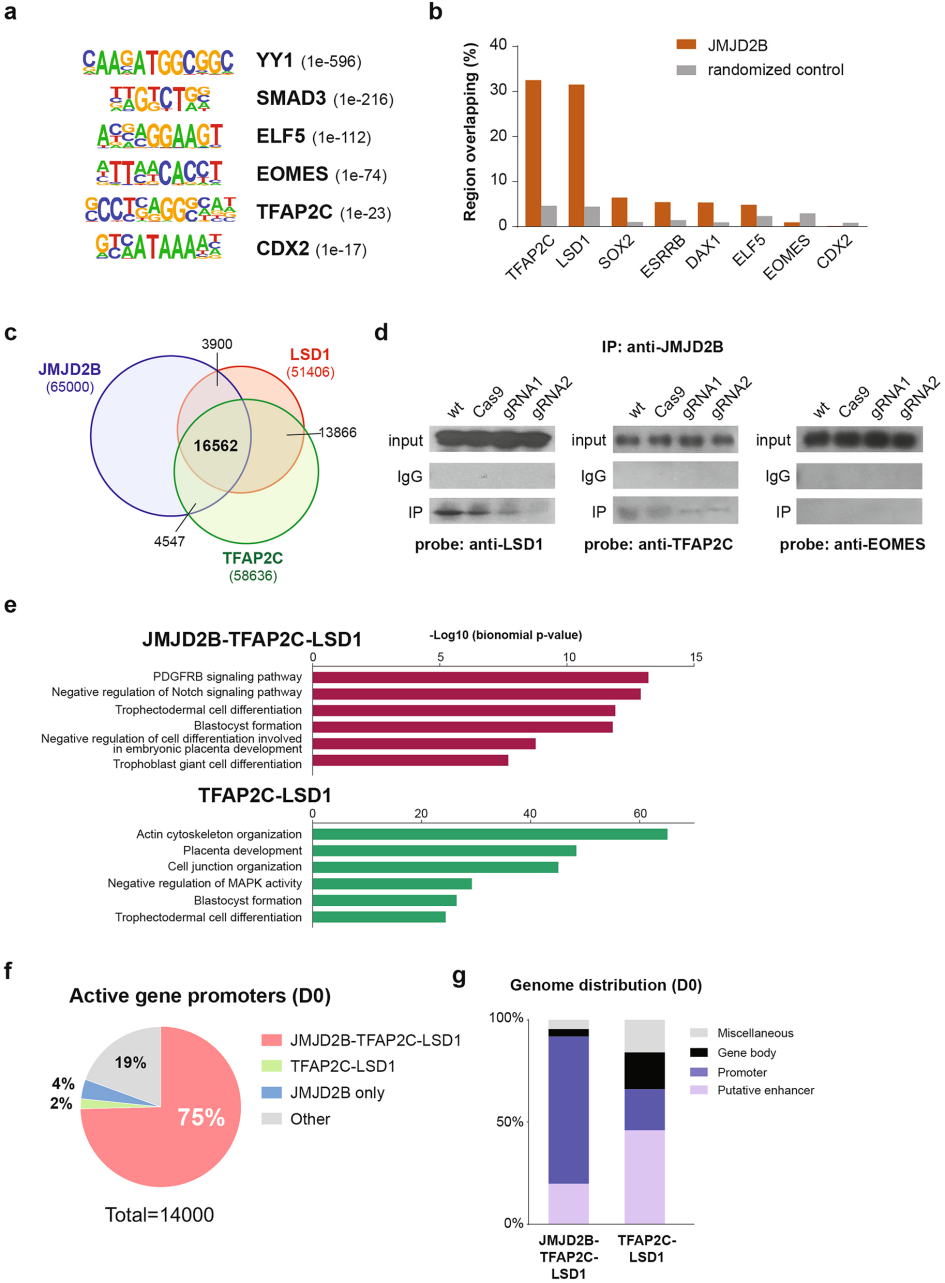
JMJD2B interacts with TFAP2C and LSD1 at active gene promoters. (**a**) Transcription factor binding motifs identified from the top 65,000 JMJD2B binding loci in undifferentiated TSCs (D0). (**b**) Bar chart showing the percentage of overlapping between JMJD2B and other TSC factors. Randomized controls of each dataset were generated from random shuffling of chromosomal regions by 10,000 times. Regions that consists of at least 1 bp intersection between the tested samples were considered as overlapped. (**c**) Venn diagram showing the number of loci occupies by JMJD2B, TFAP2C and LSD1 in TSCs. (**d**) Immunoprecipitation (IP) of JMJD2B in TSCs followed by Western blot detection using anti-LSD1, anti-TFAP2C, or anti-EOMES antibody. JMJD2B protein interaction with LSD1 and TFAP2C is observed, but not with EOMES, in the wild-type (wt) or Cas9-expressing (Cas9) TSC. The CRISPR knockdown samples (gRNA1 and 2) show reduced degree of protein interactions. (**e**) GREAT analysis of the biological processes for the JMJD2B-TFAP2C-LSD1 or TFAP2C-LSD1 bound loci in the undifferentiated TSCs. (**f**) Pie chart showing the occupancy of different protein complexes at the promoter of active transcribing genes in undifferentiated TSCs. (**g**) Genome-wide distribution of the JMJD2B-TFAP2C-LSD1 and TFAP2C-LSD1 loci. Enhancers are annotated by co-enrichment of H3K4me1 and H3K27ac.

Although the presence of JMJD2B at gene bodies was negatively associated with the H3K36me3 level, its function at active gene promoters remains unclear. Interestingly, a total of 87% and 76% upregulated DEGs showed persistent or increased (from absence to presence) promoter JMJD2B binding at D2 and D6 TSC, respectively (Supplementary Fig. 6a), indicating that the presence of JMJD2B at promoters is generally associated with active gene expression in TSCs. Since the JMJD2 family functions through the binding of co-factors in gene regulation^22,35–37^, we performed motif enrichment analysis of the JMJD2B ChIP-seq peaks in undifferentiated TSCs. The JMJD2B bound loci were significantly enriched with the binding motifs of key trophoblast factors, including YY1, SMAD3, ELF5, EOMES, TFAP2C, and CDX2 (Fig. 5a). To further reveal the potential interactions between JMJD2B and other co-factors, we compared the genome-wide JMJD2B occupancy from this study with other published transcription factor binding profiles in TSCs, including CDX2, ELF5, EOMES, ESRRB, SOX2, TFAP2C, LSD1, and DAX1 datasets^8,10,38,39^. Importantly, JMJD2B bound loci were shown particularly co-occupied by the AP-2 family transcription factor TFAP2C and the histone demethylase LSD1, which are essential factors in maintaining the multipotent trophoblast compartment^6,40,41^. Other factors showed a much lower degree of overlapping with JMJD2B (Fig. 5b). We also performed comparisons with randomized control samples which were generated from random shuffling of chromosomal regions by 10,000 times (Fig. 5b), and the TFAP2C and LSD1 ChIP-seq data from other cell types^8,42,43^ (Supplementary Fig. 6b). All these negative controls showed a low degree of overlapping with our TSC JMJD2B ChIP-seq signals, indicating that the co-occupancy of JMJD2B with TFAP2C or LSD1 is highly specific in TSCs. Cross-comparison of the JMJD2B, TFAP2C, and LSD1 datasets revealed that a significant number of loci were co-occupied by these three factors, suggesting their collaborative role in gene regulation (Fig. 5c). We performed co-immunoprecipitation with the anti-JMJD2B antibody using DNase-treated TSC lysate, which eliminates physical interactions through DNA binding. It showed that JMJD2B can physically interact with LSD1 or TFAP2C proteins, but not with EOMES (Fig. 5d and Supplementary Fig. 7). Such protein–protein interactions with JMJD2B can also be demonstrated using either anti-TFAP2C or anti-LSD1 antibody for the pull-down (Supplementary Fig. 6c and 7). Strikingly, these protein interactions were not observed in the CRISPR *Jmjd2b* knockdown samples, further supporting that TFAP2C and LSD1 are the co-factors of JMJD2B.

### JMJD2B–TFAP2C–LSD1 complex activates trophoblast gene promoters

Functional annotation showed that both JMJD2B–TFAP2C–LSD1 and TFAP2C–LSD1 bound loci were associated with numerous biological terms related to trophoblast cell differentiation and placenta development (Fig. 5e). According to the transcriptome data, 75% of active genes (10,446 out of 14,000) in TSCs were bound by JMJD2B-TFAP2C-LSD1 at their promoters, whereas only 2% and 4% were occupied by TFAP2C–LSD1 and JMJD2B per se, respectively (Fig. 5f and Supplementary Table 3). The predominant occupancy of JMJD2B–TFAP2C–LSD1 at active promoters is not simply due to the fact that these factors occupied a large number of gene loci in TSCs. In fact, a substantial number of promoters (including active and silent) were not triply bound. This can be illustrated by our result showing that there were 4,452 promoters with only JMJD2B binding or 876 promoters with TFAP2C–LSD1 binding, when comparing to the 14,441 promoters with JMJD2B–TFAP2C–LSD1 binding (14,441 promoters) (Supplementary Fig. 6d). Interestingly, 68% of the TFAP2C–LSD1 promoter bound genes were silenced, as opposed to the 28% bound by the JMJD2B–TFAP2C–LSD1 complex. This suggests that the binding of JMJD2B is crucial to the activation of promoters occupied by TFAP2C and LSD1.

We noticed that a considerable number of the TFAP2C–LSD1 binding sites outside gene promoters which were not overlapped with the JMJD2B peaks. To determine the genome-wide distribution of the JMJD2B–TFAP2C–LSD1 and TFAP2C–LSD1 loci, we defined active gene enhancers based on the co-enrichment of H3K4me1 and H3K27ac using the TSC ChIP-seq datasets from Chuong et al.^38^. Among the JMJD2B–TFAP2C–LSD1 loci, 72% and 20% were located at the promoters and enhancers, respectively. However, 46% of the TFAP2C–LSD1 loci were annotated to the enhancers, with only 20% at the promoters (Fig. 5g). The occupancy of promoters and enhancers by JMJD2B–TFAP2C–LSD1 and TFAP2C–LSD1, respectively, were further illustrated in a panel of active genes (*Eomes*, *Hand1*, *Cited2*, *Fgfr1*, *Fgfr2*, *Smad6*, and *Bmp7*) (Supplementary Fig. 8). Taken together, these data suggest that the JMJD2B–TFAP2C–LSD1 and TFAP2C–LSD1 complexes have distinctive molecular functions at the gene regulatory elements of key trophoblast factors.

## Discussion

Multipotent TSC is an origin of the trophoblast lineage. Proper maintenance and differentiation of this population of cells are crucial for the formation of a functional placenta. It is known that transcriptional programs are well orchestrated by epigenetic regulations when stem cells differentiate into highly specialized cells. Although it has been demonstrated that epigenetic modulation mediated by JMJD2 histone demethylases is required for ESC maintenance and differentiation, the function of JMJD2 proteins in TSCs has not been elucidated. Here we show that JMJD2B has an essential TSC-specific role in the maintenance of the trophoblast lineage fate. Our data demonstrate that JMJD2B associates negatively with the H3K9me3 pattern in only a small subset of genes during TSC differentiation. We further provide evidence that JMJD2B forms a protein complex with TFAP2C and LSD1, which could be crucial to the activation of key trophoblast genes. Therefore, we propose that JMJD2B functions in a context-dependent manner via distinctive epigenetic targets and protein interactors.

A previous study showed that H3K9me3 was associated with the repression of several key trophoblast factors during TSC differentiation^15^, which is in line with our findings at *Cdx2* and *Eomes* loci. However, our genome-wide profiling data propose that the general role of H3K9me3 in TSCs is to safeguard the trophoblast fate by stable silencing of genes involved in other embryonic lineages. This is indeed similar to the observation in ESCs, in which the repressive histone mark H3K9me3 plays an important role in the formation of heterochromatin for the maintenance of genome stability and the transcriptional silencing of differentiation genes^44^. Depletion of *Setdb1*, a H3K9 methyltransferase, in ESCs can induce trophoblast cell fate^13,14^, presumably through derepression of *Cdx2*, which is a key upstream trophoblast fate determinant, by reducing its promoter H3K9me3 level, or through reactivation of the H3K9me3-associated endogenous retroviruses (ERVs) that are enriched at the murine TSC enhancer elements^38^. Even though H3K9me3 is associated with the expression of a few key trophoblast factors in TSCs, we notice that its modulation is independent of the histone demethylase JMJD2B. This is supported by our findings that the low H3K9me3 levels at *Cdx2* and *Eomes* remain unchanged in the *Jmjd2b-*knockdown TSCs. Interestingly, we observed a persistent co-enrichment of JMJD2B and H3K9me3 at transcriptionally silent genes during TSC differentiation. It has been reported that the *Drosophila* H3K9me3-bound Heterochromatin Protein 1a (HP1a) interacts with dKdm4a (orthologs of JMJD2A) at heterochromatin. Mutation of *dKdm4a* leads to increased enrichment of H3K36me3 at both heterochromatic and euchromatic targets^45^. Similarly, we also observe a negative correlation between JMJD2B and H3K36me3 patterns at the gene bodies of H3K9me3-associated non-trophoblast lineage genes, suggesting that JMJD2B prevents inappropriate gene activation thought its H3K36 demethylation activity. How JMJD2B functions by targeting H3K36me3 but not H3K9me3 in TSCs is not clear. Nevertheless, previous works on other histone demethylases showed that the binding of co-factors or the presence of other histone modifications can affect the substrate specificity from one histone lysine residue to another^46,47^. In addition, it is noteworthy that JMJD2 proteins show redundant functions in stem cell maintenance and differentiation. Knockout ESC lines and mouse models showed that individual JMJD2 members are dispensable for pluripotency and in vivo embryo development^19,23,48^. Nevertheless, JMJD2A and JMJD2C act together to safeguard the ESC identity by enabling transcriptional competency via constitutive removal of H3K9me3 and H3K36me3 at the active gene promoters^19^. The *Drosophila* dKdm4a and dKdm4b also acted synergistically for the prevention of developmental arrest. Only the double homozygous mutants exhibited motility at the second instar larval stage, while single knockout mutants were viable and fertile^49^. Therefore, it is likely that the H3K9me3 pattern at key trophoblast factors is regulated by multiple JMJD2 members. However, a single knockdown of *Jmjd2b* triggers TSC differentiation, which highlights the essential role of JMJD2B in the regulation of the transcriptional program governing TSC properties. It is thus important to elucidate how this JMJD2B-associated network shares common targets with other JMJD2 proteins in TSCs.

Although the presence of JMJD2B at gene promoters is generally associated with active gene expression in TSCs, we notice that a number of promoters with JMJD2B binding per se remained silent. This suggests that the JMJD2B binding does not equate at all to its activity. In fact, JMJD2 family proteins reside in large multimeric complexes and act as either transcriptional co-activators or co-repressors depending on the associated protein partners. Several protein complexes containing JMJD2 proteins have been reported, including nuclear receptor corepressor (N-CoR)^35^, androgen receptor^50,51^, MLL complex^37^, peroxisome proliferator-activated receptor gamma (PPARγ)^36^, histone deacetylases (HDACs)^52^ and polycomb repressive complex 2 (PRC2)^22^. Notably, some of the transcriptional regulations mediated by the JMJD2-containing complexes are independent of histone demethylation, but conferred with the modulation of the protein partner activity^35,36^. Our data indicate that JMJD2B interacts with TFAP2C and LSD1 as a transcriptional activator complex, which is predominantly located at active gene promoters. Previous findings on the interactome of TSCs showed that TFAP2C is one of the interacting partners of LSD1^10^. However, the function of TFAP2C–LSD1 complex remains unclear. Through the analysis of the binding patterns of TFAP2C and LSD1, we found that the TFAP2C–LSD1 complex occupies at a significant portion of inactive promoters, which is in contrast to the JMJD2B–TFAP2C–LSD1 complex. This suggests that the interaction with JMJD2B converts the protein complex into a transcriptional activator. Interestingly, we notice that a majority of the TFAP2C–LSD1 complex occupies gene enhancers. It has been reported that both TFAP2C and LSD1 are required for chromatin looping, which facilitates promoter-enhancer interaction^53,54^. Besides, TSCs with TFAP2C depletion cannot be maintained^7^, whereas LSD1-depleted TSCs are prone to differentiate^41^. LSD1 also occupies active enhancers in undifferentiated ESCs and its demethylase activity is inhibited by the presence of acetylated histones^55,56^. During ESC differentiation, enhancer histone acetylation is decreased owing to the reduced occupancy of the OCT4-p300 complex, which subsequently permits H3K4 demethylation by LSD1. Therefore, we speculate that JMJD2B may facilitate long-range chromatin interactions with the TFAP2C–LSD1 containing complexes, and possibly modulate its activity, to maintain the stemness transcriptional program in TSCs.

**Figure 6:**
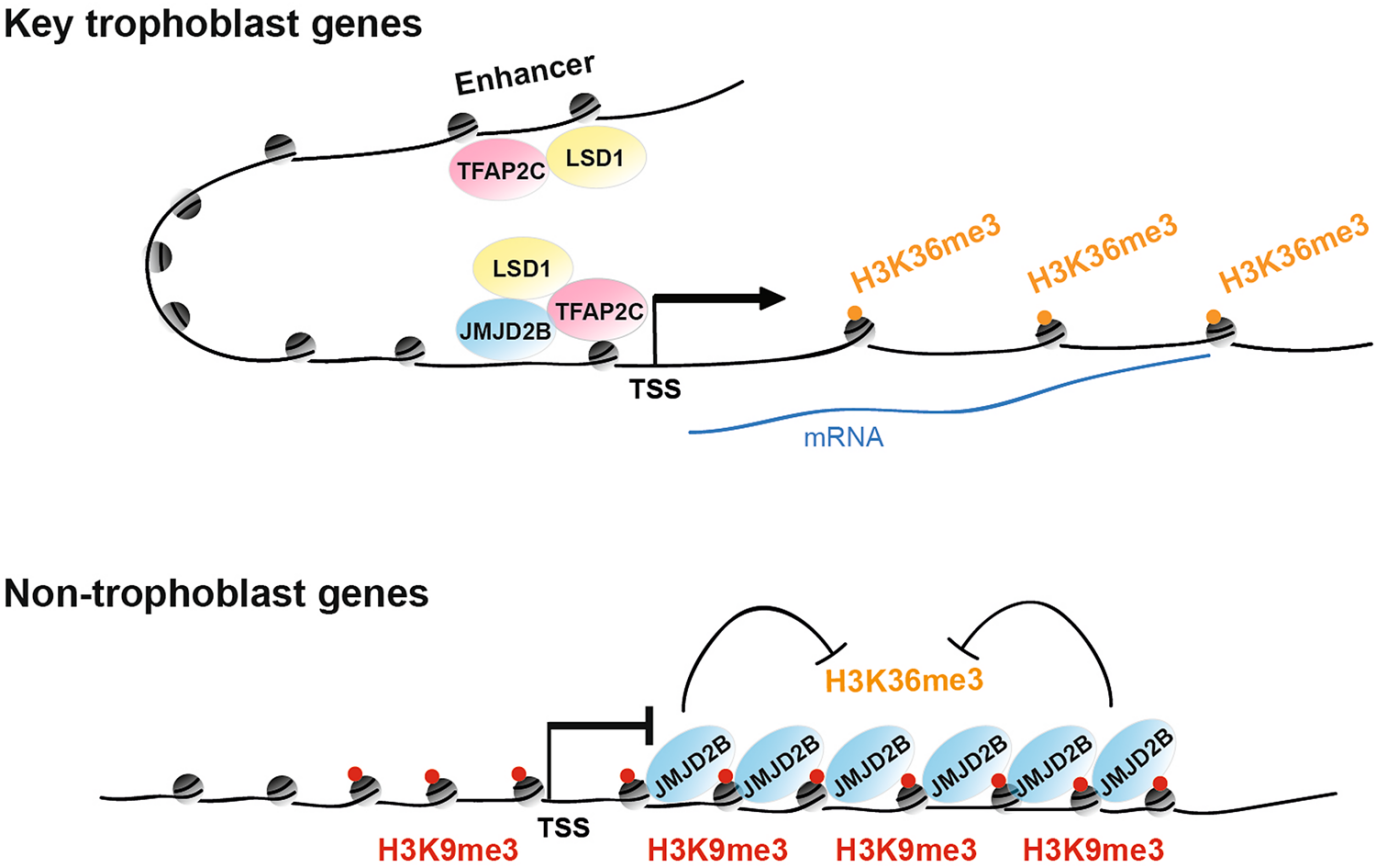
A model of the dual function of JMJD2B for the transcriptional regulation in TSCs. At the promoters of key trophoblast genes, JMJD2B forms a transcriptional activator complex with TFAP2C and LSD1, which may interact with the TFAP2C–LSD1 complex at enhancer, to maintain active gene expression. At the gene bodies of non-trophoblast genes, JMJD2B is co-enriched with the repressive H3K9me3 mark, and mediates H3K36 demethylation for silencing of unrelated lineage fate. The drawing was created originally using Microsoft PowerPoint.

In summary, we propose a model for the dual function of JMJD2B in safeguarding the TSC identity (Fig. 6). JMJD2B forms a protein complex with TFAP2C and LSD1 at the promoters of key trophoblast genes to maintain active gene expression, presumably through mediating promoter-enhancer interactions. On the other hand, JMJD2B is recruited by the H3K9me3 mark at the non-trophoblast lineage genes and represses their expression through H3K36 demethylation. Our work thus uncovers the crosstalk between epigenetic modulators and transcription factors, which provides novel insights into the epigenetic mechanism in trophoblast development.

## Methods

### Cell culture

TSC line was derived from ROSA26 (129 Sv) mice by Dr. Rossant group^57^ and cultured in TS basic medium (RPMI1640 medium (Gibco, CA, USA) with 20% fetal bovine serum (HyClone, MA, USA), 1 mM sodium pyruvate (Gibco), 1 × penicillin–streptomycin-glutamine (Gibco), and 50 μM β-mercaptoethanol (Sigma, MO, USA), 25 ng/mL hFGF4 (Peprotech, NJ, USA) and 1 μg/mL heparin (Sigma)), with 70% medium pre-conditioned on embryonic fibroblasts^57^. TSC differentiation medium consisted of the TS basic medium without hFGF4 and heparin. J1B-04 (Tocris Bioscience, MN, USA) was prepared in DMSO and stored at − 20 °C as a stock solution. TSCs were cultured at 37 °C with 5% CO_2_. The culture medium was changed every 2 days.

### Jmjd2 gene knockdown

TSCs were transfected with 62.5 nM double-stranded siRNA (GenePharma, China) using TransIT-X2 (Mirus Bio, WI, USA) according to the manufacturer’s instructions. Samples were analyzed 72 h after transfection. For CRISPR-Cas9 genome editing, TSCs were transduced by lentivirus generated from the LentiCRISPRv2-mCherry plasmid. The mCherry positive Cas9-expressing TSCs were isolated by fluorescence activated cell sorting (FACS) using BD FACSAria II cell sorter (BD Bioscience, CA USA). Two Alt-R CRISPR-Cas9 crRNAs (IDT, IA, USA), which targeted the *Jmjd2b* genomic sequence, were synthesized and separately annealed with the Alt-R CRISPR-Cas9 tracrRNA (IDT) to form the duplexed oligo. The Cas9-expressing TSCs were transfected with 90 nM duplexed oligo using TransIT-X2 (Mirus Bio). Two *Jmjd2b-*knockdown TSC lines were selected and verified by T7 endonuclease I digestion and Sanger sequencing (Supplementary Fig. 9). The *Jmjd2b-*knockdown TSCs were used for other experiments within 5 passages. The siRNA and crRNA sequences were listed in Supplementary Table 4.

### RNA extraction and quantitative RT-PCR (qRT-PCR)

Total RNA was extracted using MiniBEST Universal RNA Extraction Kit (Takara, Japan) and reverse transcribed by Transcriptor First Strand cDNA Synthesis Kit (Roche, Germany) according to the manufacturer’s instructions. qRT-PCR was performed on a StepOnePlus System (Thermo Fisher Scientific, MA, USA) using the SYBR Premix Ex Taq (Takara) and primers listed in Supplementary Table 4. Cycle threshold (Ct) values of target genes were normalized against the housekeeping gene *Dynein* or *β-Actin*. All samples were analyzed in duplicates and three independent experiments were performed.

### Immunocytochemistry staining

TSCs seeded on gelatin-coated coverslips were fixed with 4% paraformaldehyde for 10 min at room temperature. Cells were permeabilized by 0.1% Triton X-100 for 10 min. Immunostainings were performed after blocking in 1% BSA for 1 h using the following antibodies and dilutions: anti-EOMES (Abcam, UK, ab23345) 1:250; anti-ASCL2 (Millipore, MA, USA, MAB4418) 1:200; anti-HAND1 (Santa Cruz Biotechnology, TX, USA, sc-390376) 1:100. Secondary antibodies were anti-IgG conjugated with Alexa Fluor 555 (Invitrogen, CA, USA). Cells were stained with 0.1 µg/ml DAPI for 1 min. Photographs were taken on an Olympus BX53 epifluorescence microscope at 400X magnification with the following exposure time: 18 ms for DAPI; 400 ms for EOMES; 500 ms for ASCL2; 700 ms for HAND1.

### Chromatin immunoprecipitation (ChIP)

ChIP was performed with anti-Jmjd2b (Abcam ab191434), anti-H3K9me3 (Abcam ab8898), or anti-H3K36me3 (Abcam ab9050) using MAGnify Chromatin Immunoprecipitation System (Invitrogen) according to the manufacturer’s instructions. Chromatin was sonicated by Bioruptor Plus UCD-300 (Diagenode, Belgium). ChIP with IgG was served as a negative control. Immunoprecipitated DNA was analyzed by qPCR with the relevant primers listed in Supplementary Table 4. Enrichment level was calculated as a percentage of input. Three independent experiments were performed.

### Western blotting

Cells were lysed in lysis buffer containing 50 mM Tris–HCl pH6.6, 150 mM NaCl, 1% Triton X-100, 0.1% SDS, 0.1% Na deoxycholate and 1% NP-40 and 1 × complete protease inhibitor cocktail (Roche), 1 μg/ml antipain, 1 μg/ml leupeptin, 1 mg/ml pepstatin, 2 μg/ ml aprotinin, 1 mM PMSF on ice for 30 min. The cell lysates were loaded on a 8–10% SDS–polyacrylamide gel for SDS-PAGE, followed by electro-transfer to a PVDF membrane. The membrane was blocked with 5% skim milk in TBS-Tween-20 (0.1%, v/v) for 2 h and then incubated with either anti-GAPDH (Cell Signaling Technology, MA, USA, #2118, 1:1000), anti-JMJD2A (Cell Signaling Technology #5328, 1:1000), anti-JMJD2B (Abcam ab191434, 1:1000), or anti-JMJD2C (Novus Biologicals, CO, USA, NB110-38884, 1:1000) at 4 °C overnight, followed by incubation with horseradish peroxidase-conjugated anti-rabbit IgG secondary antibody. Immunoreactive protein was visualized using ECL prime solution (GE Healthcare, IL, USA).

### Co-immunoprecipitation

TSCs were lysed in 100 μL ice-cold non-denaturing lysis buffer (50 mM Tris–HCl pH7.4, 150 mM NaCl, 1 mM EDTA, 1% NP-40, 1 μg/ml antipain, 1 μg/ml leupeptin, 1 mg/ml pepstatin, 2 μg/ml aprotinin, 1 mM PMSF) with 5U DNase (Thermo Fisher Scientific) at 37 °C for 15 min and then on ice for 90 min. Anti-JMJD2B antibody (Abcam ab191434), anti-LSD1 (Abcam ab17721), or anti-TFAP2C antibody (Santa Cruz Biotechnology sc-12762) was conjugated with Dynabeads Protein A/G beads (Invitrogen) and mixed with 1 mg protein lysate at 4 °C overnight. The Dynabeads-antibody-antigen complex was washed 3 times with PBS for 5 min at 4 °C. The immunoprecipitated protein was eluted in protein loading buffer (2% SDS, 2% beta-mercaptoethanol, 4% glycerol, 0.04 M Tris–HCl pH 6.8, 0.01% bromophenol blue) at 95 °C for 10 min. The eluted proteins were subjected to Western blotting with anti-JMJD2B (Abcam ab191434, 1:1000), anti-LSD1 antibody (Abcam ab17721, 1:1000), anti-TFAP2C antibody (Santa Cruz Biotechnology sc-12762, 1:1000) or anti-EOMES (Abcam ab23345, 1:1000).

### RNA-sequencing (RNA-seq) analysis

Total RNA sample was used to construct sequencing library using KAPA Stranded mRNA-Seq kit (KAPA Biosystems, MA, USA), followed by paired-end sequencing using HiSeq 1500/2000 (Illumina, CA, USA) at the Center for Genomic Sciences (University of Hong Kong). Two biological replicates of each time point sample were subjected to RNA-seq. The raw sequencing reads were filtered against low quality bases (quality < 20), partial Illumina adaptor sequences, and rRNA/tRNA contamination by Trimmomatics and Bowtie2. Read alignment against *Mus musculus* mm10 (Ensembl release 89) genomic assembly was performed using Tophat2. Duplicate reads were removed by MarkDuplicates from Picard (Broad Institute). Transcriptome assembly and differential gene expression were calculated in fragments per kilobase of transcript per million mapped reads (FPKM) unit by Cufflinks. Differentially expressed genes (DEGs) were selected with more than 1.5-fold changes in FPKM values between samples. To determine gene transcriptional status, zFPKM transformation was performed using R package. zFPKM value over − 3 was regarded as transcriptionally active genes as recommended by Hart et al.^33^. Gene ontology (GO) analysis was performed using the two-side hypergeometric test from the ClueGO plugin of Cytoscape software. Enriched GO terms with Benjamini–Hochberg FDR-corrected q-value < 0.05 were selected.

### ChIP-sequencing (ChIP-seq) analysis

ChIP DNA fragments were used to construct sequencing library using MicroPlex Library Preparation Kit v2 (Diagenode), followed by paired-end sequencing using HiSeq 1500/2000 (Illumina, CA, USA) at the Center for Genomic Sciences (University of Hong Kong). Two biological replicates of each time point sample were subjected to ChIP-seq. Raw reads were processed as described in the RNA-seq analysis section. Peak calling and differential binding events were analyzed by MACS2 using the default cutoff value (0.1) of FDR-corrected q-value. Top 65,000 statistically significant peaks (sorted by FDR-corrected q-value) were selected for all the ChIP-seq datasets, which is corresponding to a mean FDR-corrected q-value cutoff at 3 × 10^–4^. Signal density heatmap and profile of ChIP-Seq signals were generated by deepTools. Scores per genome regions were calculated by ComputeMatrix and then utilized by plotProfile and plotHeatmap for graph plotting. The GO biological processes and PANTHER pathway analyses were conducted using Genomic Regions Enrichment of Annotations Tool (GREAT). The threshold for region-based enrichment term was set twofold for GO biological processes and onefold for PANTHER pathway analysis. Enriched GO terms with Benjamini–Hochberg FDR-corrected p-value < 0.05 were selected. Motif analysis was performed using HOMER. Published TSC ChIP-seq datasets were obtained from European Nucleotide Archive: H3K4me1, H3K27ac, CDX2, EOMES, and ELF5 (PRJNA179294)^38^; H3K36me3 (PRJNA170844)^34^; TFAP2C (PRJNA298763)^10^; SOX2 (PRJNA139231)^8^; LSD1, ESRRB, and DAX1 (PRJEB9375)^39^. ChIP-seq datasets from non-TSCs: TFAP2C (PRJNA232935)^8^; LSD1 (PRJNA174296)^42^ (PRJNA219405)^43^. All the peaks with mm9 coordinates were converted to mm10 by UCSC LiftOver tool. Promoter was defined as − 3 kb to + 500 bp relative to the transcriptional start site (TSS); gene body was defined by Ensembl annotation (GRCm38.89); putative enhancer was defined by the H3K4me1 and H3K27ac overlapped regions outside the promoters. Overlapping analyses of the genomic regions were performed using Bedtools (version 2.26.0). Regions that consist of at least 1 bp intersection between the tested samples were considered as overlapped. Randomized controls were generated by shuffling peaks within the same chromosomes using Bedtools. Control peaks of individual shuffled ChIP-Seq datasets were intersected with the JMJ2B target regions and the maximum total number of intersected target regions was counted from 10,000 shuffling times.

### Statistical analysis

A statistical significant difference was calculated by two-tailed unpaired Student’s t-test or one-way ANOVA with Tukey’s test.

## Supplementary Information

Supplementary Information 1.

Supplementary Information 2.

Supplementary Information 3.

Supplementary Information 4.

## Data Availability

The RNA-seq and ChIP-seq data have been deposited in the NCBI Sequence Read Archive (SRA) under accession PRJNA524074.
