# Supplementary material for: Histone demethylase JMJD2B/KDM4B regulates transcriptional program via distinctive epigenetic targets and protein interactors for the maintenance of trophoblast stem cells
Source: Sci Rep. 2021 Jan 13;11:884. doi: 10.1038/s41598-020-79601-7 (PMC7806742; doi:10.1038/s41598-020-79601-7)
Supplement: Supplementary file 1 — Supplementary Information 1. [file 41598_2020_79601_MOESM1_ESM.pdf]

Histone demethylase JMJD2B/KDM4B regulates transcriptional program via distinctive epigenetic targets and protein interactors for the maintenance of trophoblast stem cells

Kylie Hin-Man Mak[1], Yuk Man Lam[1], Ray Kit Ng[1*]

[1] School of Biomedical Sciences, Li Ka Shing Faculty of Medicine, The University of Hong Kong, Pokfulam, Hong Kong SAR, China

* Corresponding Author: Ray Kit Ng
School of Biomedical Sciences,
Li Ka Shing Faculty of Medicine,
The University of Hong Kong
Hong Kong SAR, China
Tel: +852 39179541
Fax: +852 28551254
Email: *raykitng@hku.hk*

Supplementary Figure 1

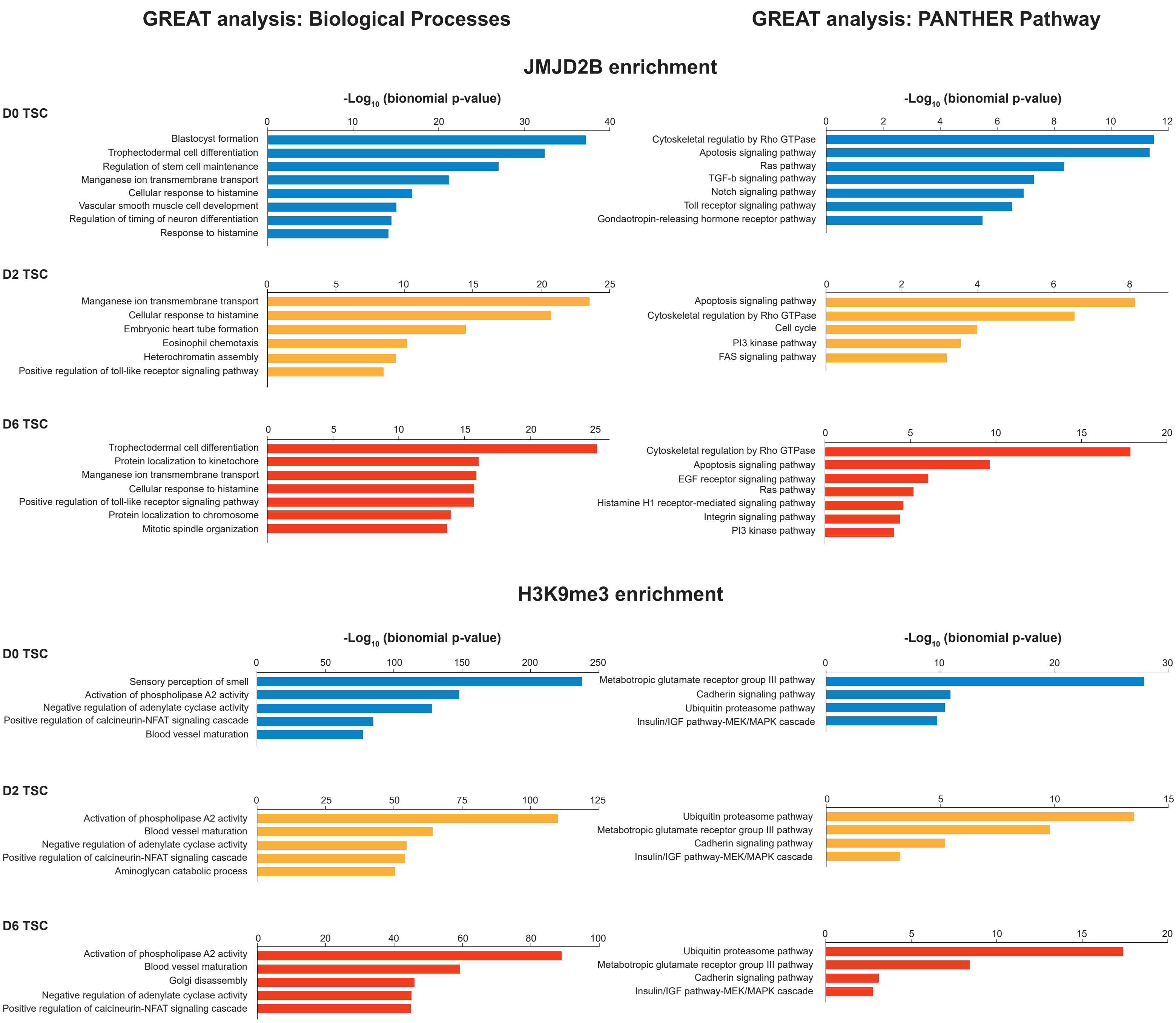

Supplementary Figure 2

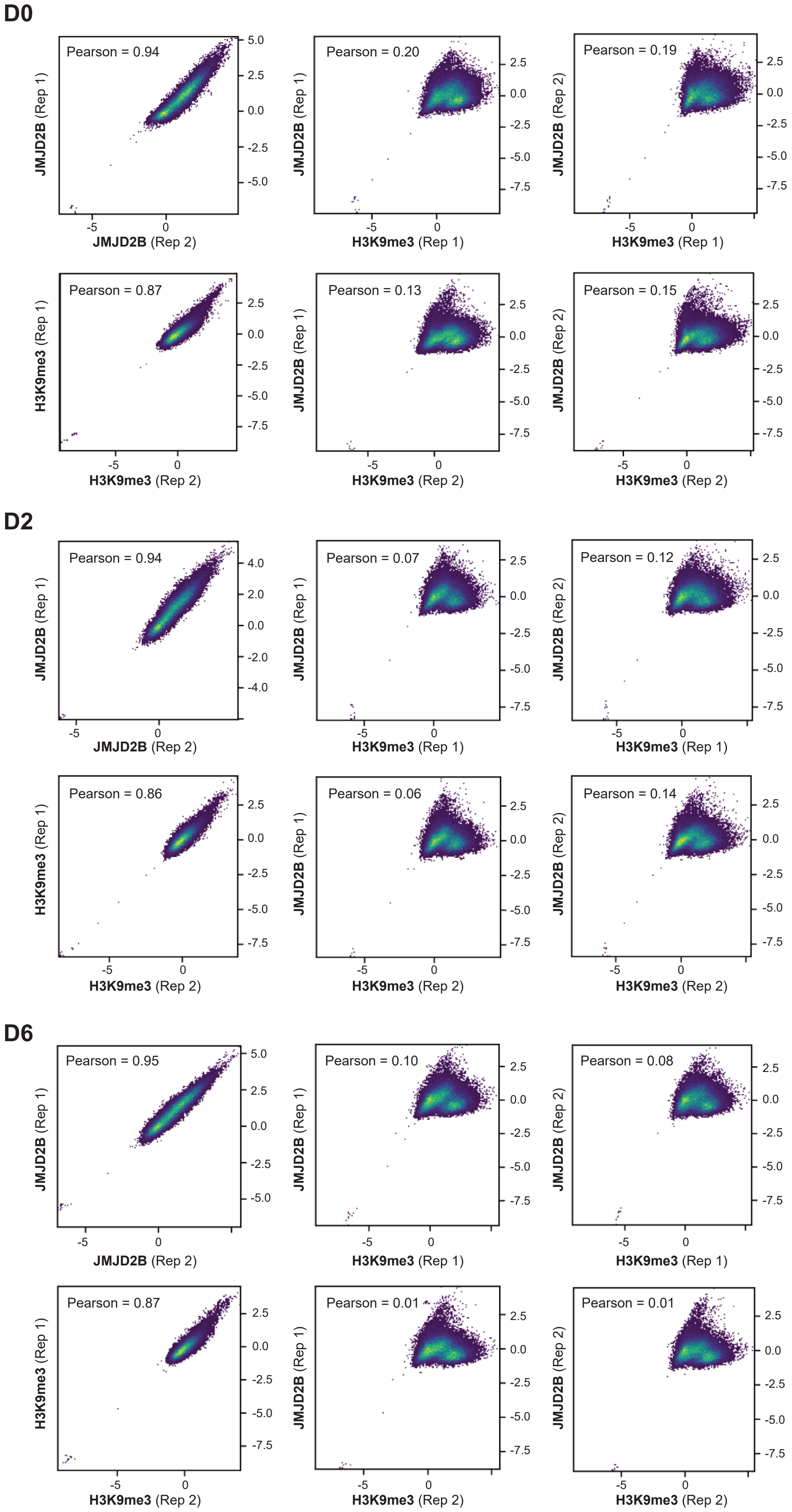

Supplementary Figure 3

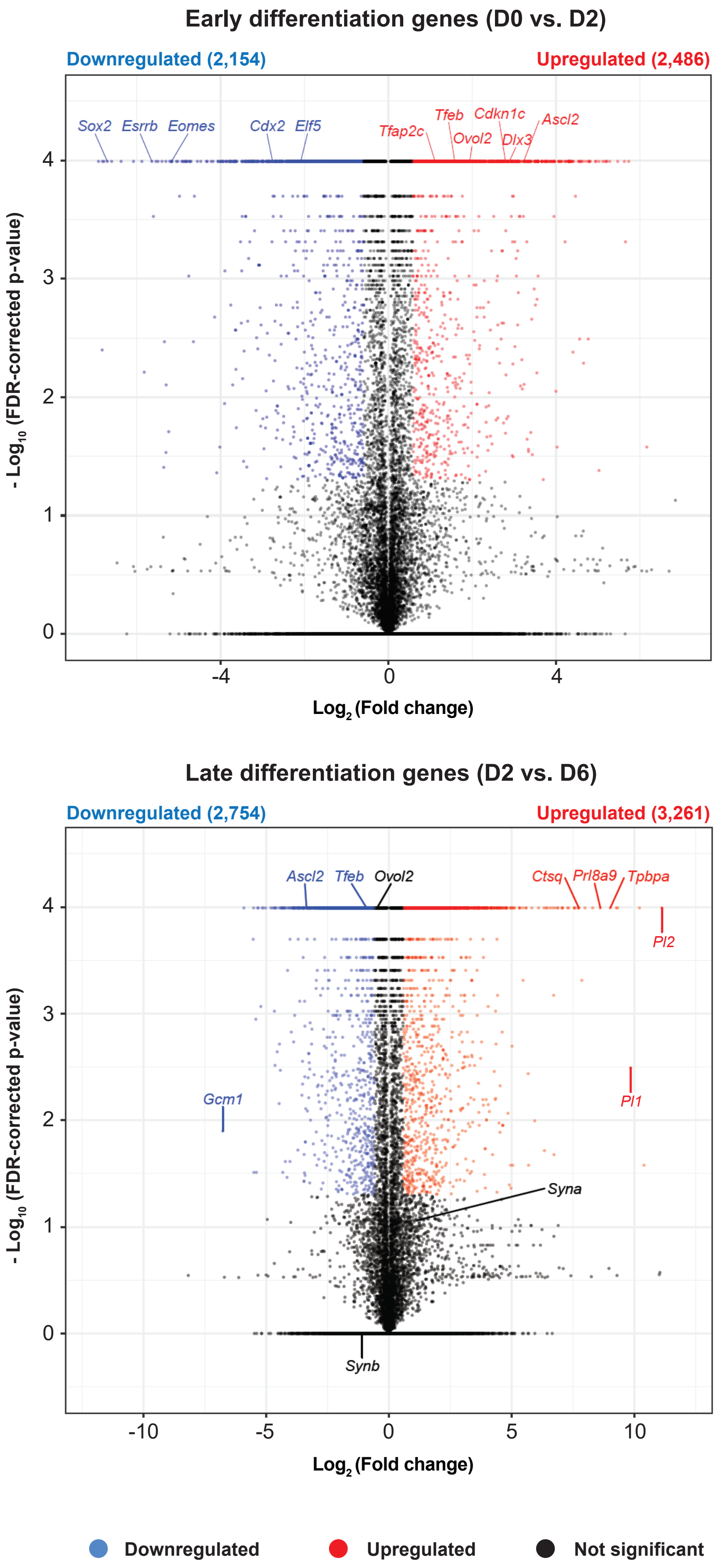

Supplementary Figure 4

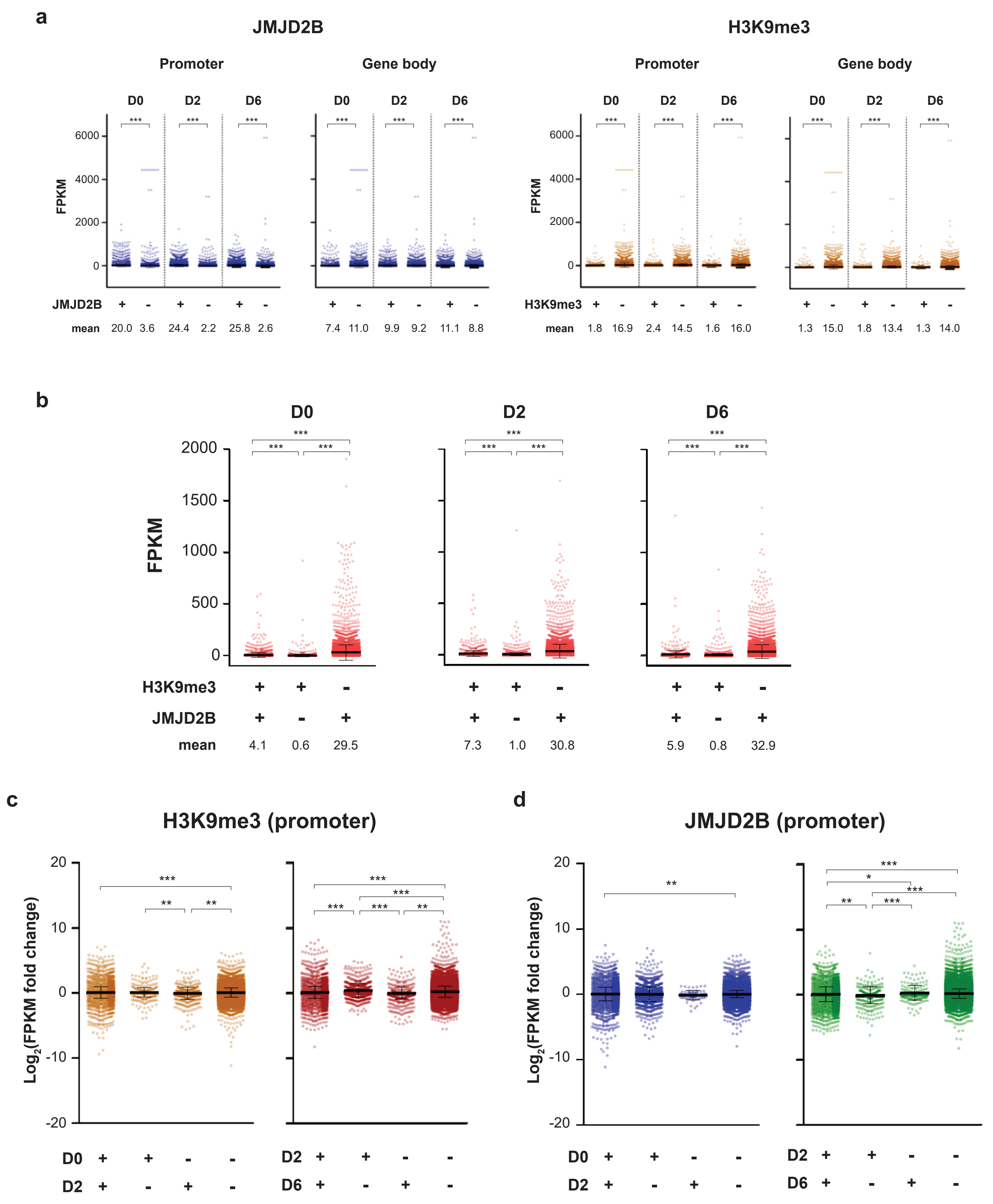

Supplementary Figure 5

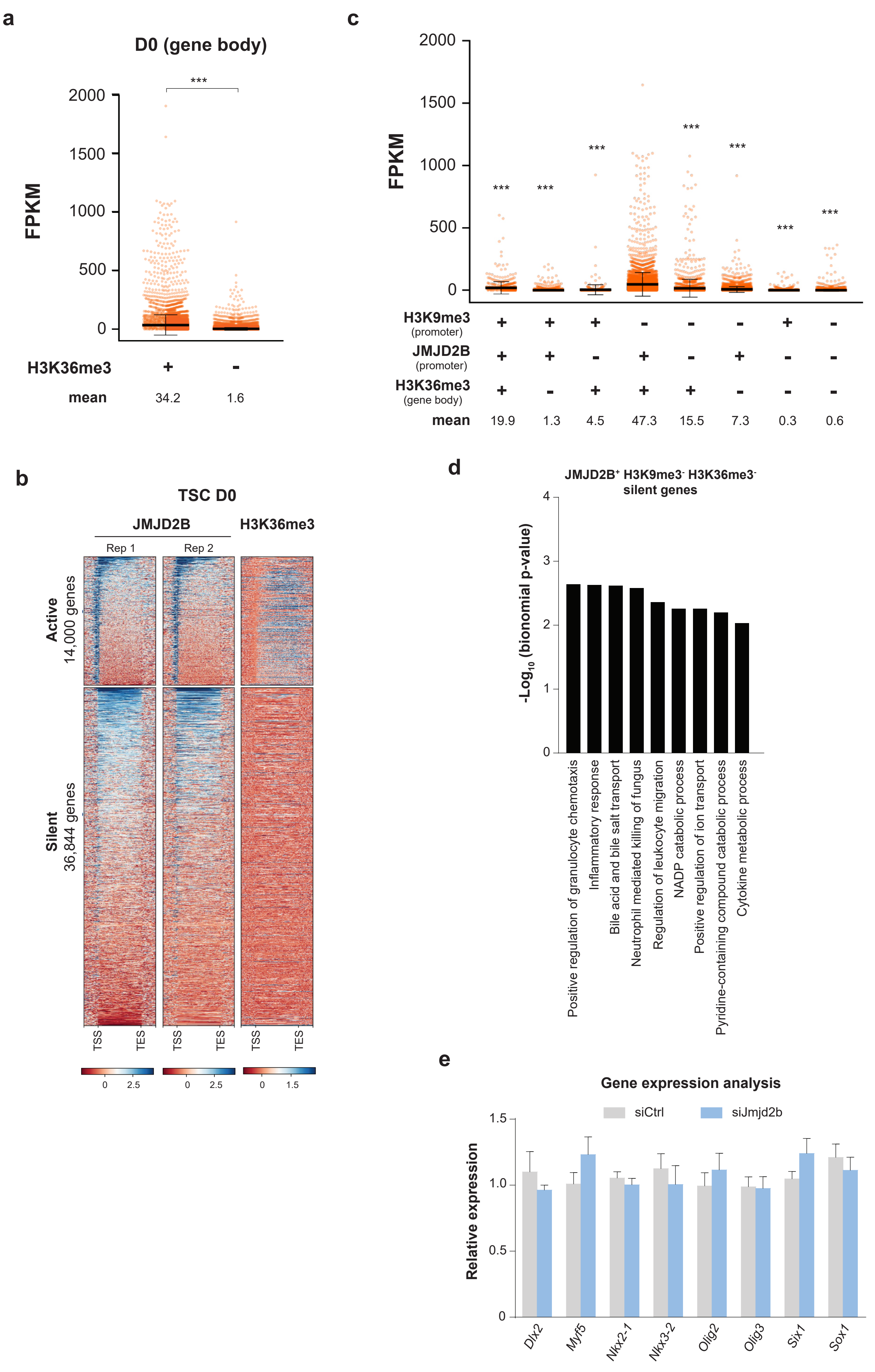

Supplementary Figure 6

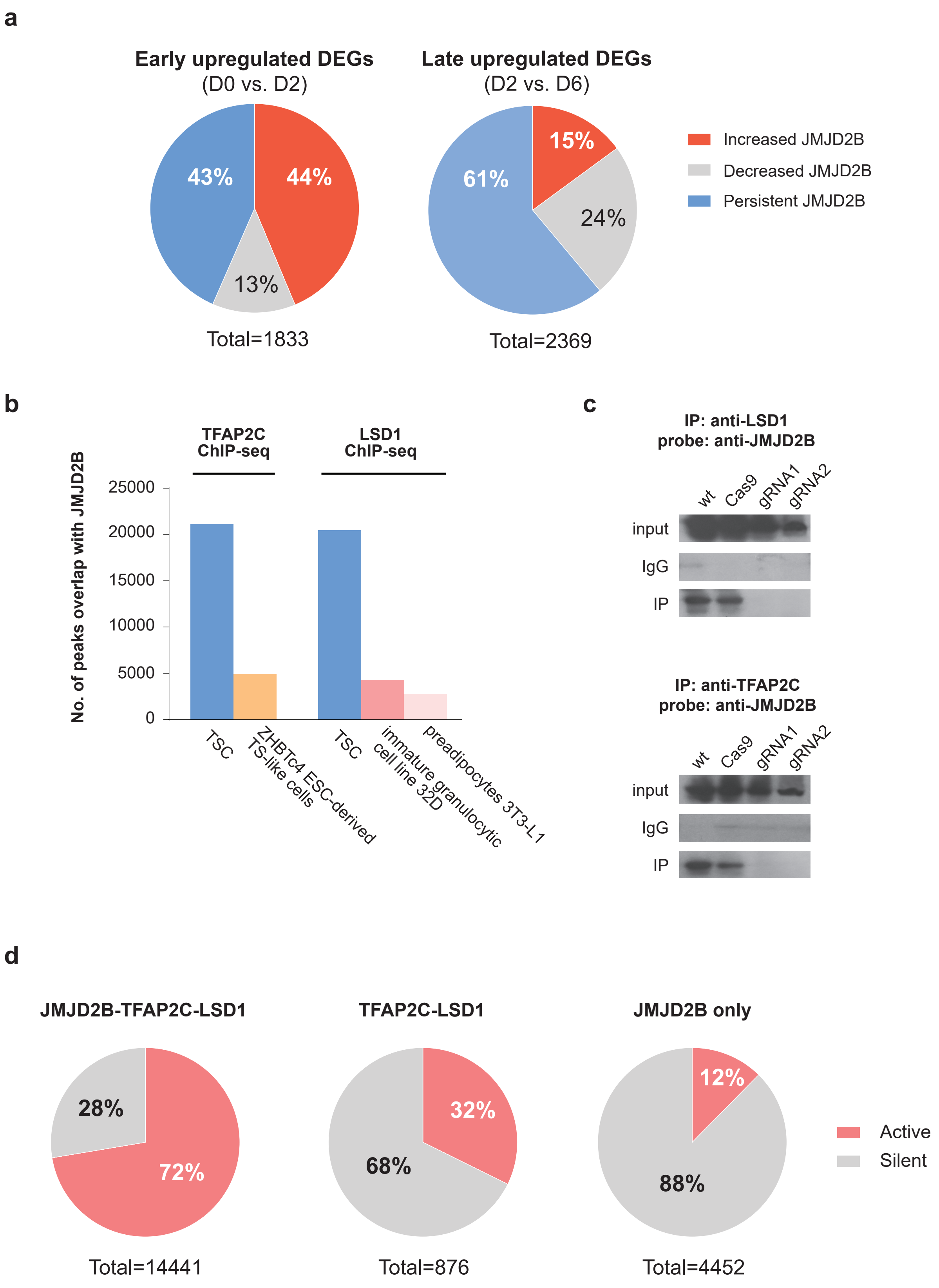

Supplementary Figure 7

(Figure 5d left)

IP: anti-JMJD2B
probe: anti-LSD1

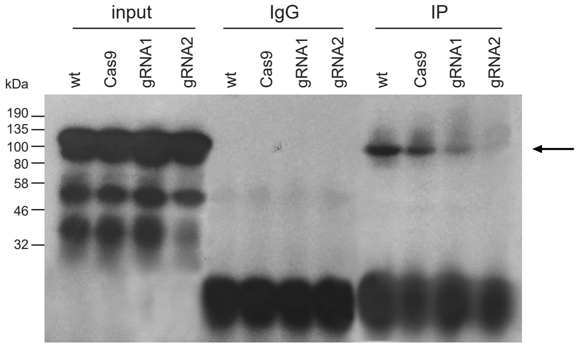

(Figure 5d middle)

IP: anti-JMJD2B
probe: anti-TFAP2C

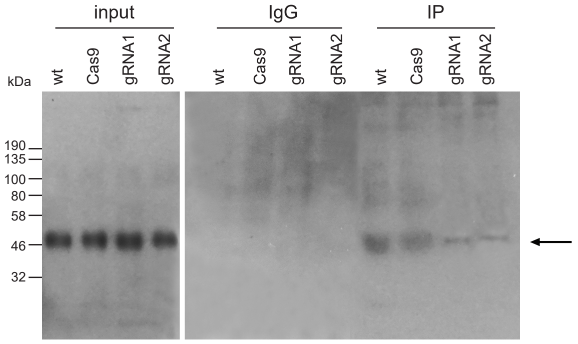

(Supplementary Figure 6c top)

IP: anti-LSD1
probe: anti-JMJD2B

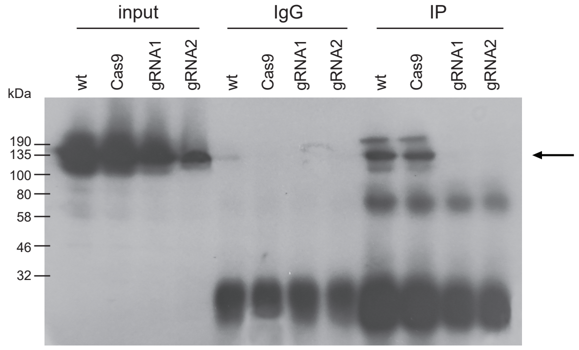

(Supplementary Figure 6c bottom)

IP: anti-TFAP2C
probe: anti-JMJD2B

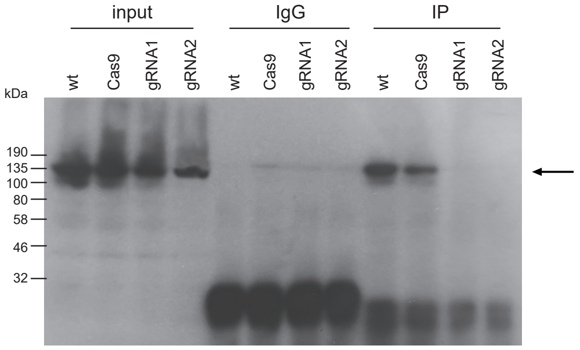

(Figure 5d right)

IP: anti-JMJD2B
probe: anti-EOMES

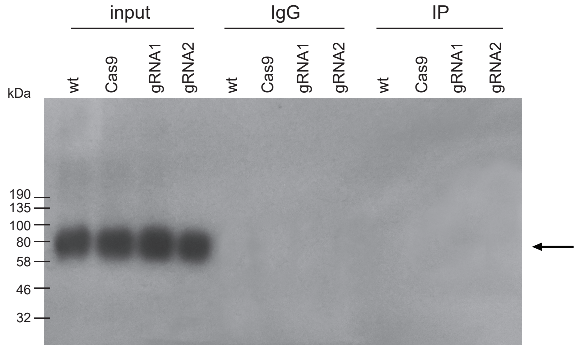

Supplementary Figure 8

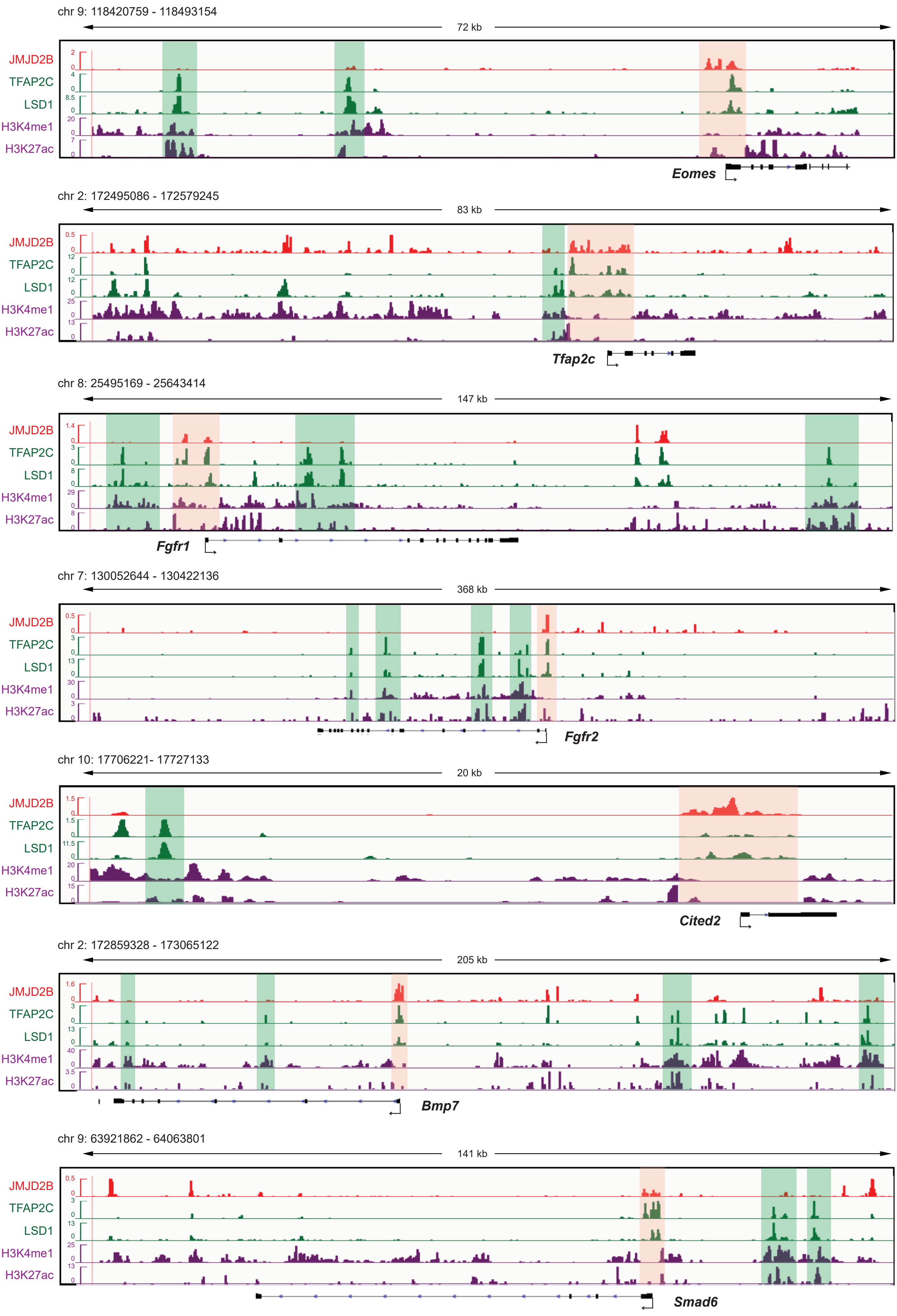

Supplementary Figure 9

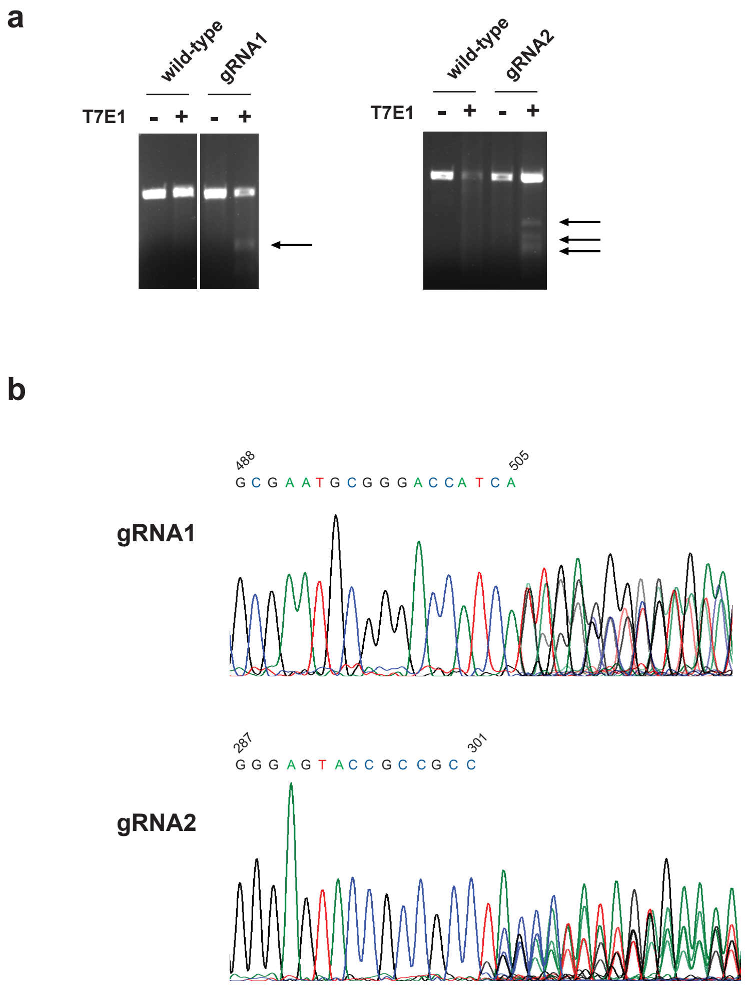

Supplementary figure legends

Supplementary Figure 1. Functional annotation of the genomic loci during *in vitro* TSC differentiation. GREAT analysis of the biological processes and PANTHER pathways for regions enriched with JMJD2B or H3K9me3 in undifferentiated (D0), 2-day differentiated (D2), and 6-day differentiated (D6) TSCs.

Supplementary Figure 2. Global correlation between JMJD2B and H3K9me3 ChIP-seq signals at gene promoters (-3kb – +500bp). Scatter plots with Pearson correlation of the JMJD2B and H3K9me3 ChIP-seq replicates of D0, D2, and D6 TSC samples.

Supplementary Figure 3. Transcriptome analysis of the *in vitro* differentiation of TSCs by RNA-seq. Volcano plots showing the early (D0 vs. D2) and late (D2 vs. D6) differentiation genes with at least 1.5-fold changes in FPKM values between samples. Upregulated genes and downregulated genes are shown in red and blue color, respectively. Genes with FDR-corrected p-value > 0.05 are regarded as not significant (black). Names of key trophoblast marker genes are annotated.

Supplementary Figure 4. Global correlation between JMJD2B, H3K9me3, and gene expression patterns in TSCs. **(a)** Dot plots showing the expression of genes with either JMJD2B or H3K9me3 enrichment at promoters or gene bodies in D0, D2, and D6 TSCs. **(b)** Expression levels of genes with different combinations of JMDJ2B and H3K9me3 patterns at promoters. **(c – d)** A global view of the changes of gene expression during TSC differentiation in association with

the changes of H3K9me3 or JMJD2B binding patterns. One-way ANOVA with Tukey's test *P < 0.05, **P < 0.01, and ***P < 0.001.

Supplementary Figure 5. Global correlation between JMJD2B and H3K36me3 in undifferentiated TSCs. **(a)** Dot plots showing the higher expression of genes with H3K36me3 mark at gene bodies in D0 TSCs. One-way ANOVA with Tukey's test ***P < 0.001. **(b)** Heatmaps showing JMJD2B and H3K36me3 enrichment levels at transcriptionally active or silent gene loci in undifferentiated TSCs. TSS: transcriptional start site; TES: transcriptional end site. **(c)** Expression levels of genes with different combinations of JMDJ2B, H3K9me3, and H3K36me3 patterns. Statistical calculation was compared to the JMJD2B^{+} H3K9me3^{-} H3K36me3^{+} group, which shows the highest expression value. One-way ANOVA with Tukey's test ***P < 0.001. **(d)** GO analysis of the biological processes for the JMJD2B^{+} H3K9me3^{-} H3K36me3^{-} silent genes in TSCs. **(c)** qRT-PCR analysis shows no significant alterations of embryonic lineage gene expression upon knockdown of *Jmjd2b*. Experiments were performed with three replicates. Data represent mean ± SD.

Supplementary Figure 6. Promoter JMJD2B binding maintains active gene transcription. **(a)** Pie charts showing the JMJD2B binding patterns at the promoters of early or late upregulated DEGs during TSC differentiation. Persistent or increased JMJD2B binding in differentiated TSCs maintains the active gene state in a majority of upregulated DEGs. "Increased" is defined by "from absence to presence"; "decreased" is defined by "from presence to absence". **(b)** Bar chart showing the percentage of overlapping between JMJD2B and TFAP2C or LSD1 ChIP-seq data from TSCs or other cell types. Regions that consist of at least 1bp intersection between the tested samples

were considered as overlapped. **(c)** Immunoprecipitation (IP) of LSD1 or TFAP2C in TSCs followed by Western blot detection using anti-JMJD2B antibody. LSD1-JMJD2B and TFAP2C-JMJD2B protein interactions are observed in the wild-type (wt) or Cas9-expressing (Cas9) TSC. The CRISPR knockdown samples (gRNA1 and 2) show a reduced degree of protein interactions. **(d)** Pie charts showing the association between transcriptional state and promoter occupancy by different protein complexes. Gene promoters bound by JMJD2B-TFAP2C-LSD1 complex are predominantly associated with active gene transcription, where those bound by TFAP2C-LSD1 complex or JMJD2B *per se* are predominantly associated with silent gene state.

Supplementary Figure 7. Original images of the co-IP experiments shown in Figure 5d and Supplementary Figure 6c. Arrows indicate the target protein band.

Supplementary Figure 8. JMJD2B-TFAP2C-LSD1 and TFAP2C-LSD1 occupancy at a panel of key TSC gene loci. ChIP-seq signal profiles showing the promoter regions that have JMJD2B-TFAP2C-LSD1 binding are highlighted in red color, while the enhancer regions that have TFAP2C-LSD1 binding are highlighted in green color. Putative enhancers are annotated by co-enrichment of H3K4me1 and H3K27ac.

Supplementary Figure 9. Verification of the CRISPR *Jmjd2b*-knockdown TSC lines. **(a)** Cleavage PCR products by T7 endonuclease I (T7E1) digestion shows the presence of mismatch nucleotides at the gRNA targeting region in *Jmjd2b* locus. **(b)** Sanger sequencing result of the targeted *Jmjd2b* locus by gRNA 1 and 2, showing that the *Jmjd2b*-knockdown lines are polyclonal.

Supplementary Table 4. List of oligo sequences

Target	Forward (5' to 3')	Reverse (5' to 3')	Application
siControl	TTCTCCGAACGTGTCACGTTT		siRNA knockdown
siJmjd2a	CGAACAUCCUACGACGAUA		
siJmjd2b	GCCCAUUAUCCCAAUGCUGUA		
siJmjd2c	GGUUCUAAGCAUUGACGAA		
gRNA1	GAATGCGGGACCATCATTGA		CRISPR-Cas9
gRNA2	ACTTTTCACTATTGGCTAGG		
T7E1-gRNA1	ATGGACGTCACTGCCAAGTT	TGAGCTGTCACCTTGTGCTC	T7E1 digestion assay
T7E1-gRNA2	GAGCCGAGCCGTGTATGAG	CAGTGCAGTGGTTCGGGTTA	
Cdx2	AGTGAGCTGGCTGCCACACT	GCTGCTGCTGCTTCTTCTTGA	Gene expression
Eomes	TCAGAGACACAGTTCATCGCTGT	CCGTGTACATGGAATCGTAGTTGT	
Elf5	CAATGATGCTGAAGAGACCAAGACT	GAGGCTTGTTCGGCTGTGAC	
Ascl2	GAGAGCTAAGCCCGATGGA	AGGTCCACCAGGAGTCACC	
Lhx2	GCATCTACTGCAAAGAAGACTACTACA	CGCATCACCATCTCTGAGG	
Hand1	CAAGCGGAAAAGGGAGTTG	GTGCGCCCTTTAATCCTCTT	
Pl1	ACAGAACACTTCCCCTGTGTC	TTGCTTTCAGAAGGTCTTCAGTT	
Dlx2	GGCCTTTCTGGGAAACTACC	TCTGCGAAGGATGCAGAAG	
Myf5	GGGCAGAATACGTGCTTTTC	GCAAAAAGAACAGGCAGAGG	
Nkx2-1	ATCTGGCCAGCATGATTCAC	TTCATCTTGTAGCGGTGGTTC	
Nkx2-3	TTAAGAACCGTCGCTACAAG	CTTTACGGCCACTTTCTTGG	
Olig2	GTGGCTTCAAGTCATCTTCCTC	GGCTCAGTCATCTGCTTCTTG	
Olig3	AGATCGCCACTCTGCTTCTG	CTCTCCAACCAACCTCTTCATC	
Six1	TTTCCCCTCCCACTAAAAGG	TGTAAAGGATGCCTGGGTTG	
Sox1	TGAAGGAACACCCGGATTAC	CAGCGAGTACTTGTCCTTCTTG	
Jmjd2a	CTGAAGAATGGATGTGCTCTCG	TGTCGTCATTGGCTCTCTGC	
Jmjd2b	ACGATGGCTCCTACAGTGACAA	GATTACCGTCTGTCCACCGAA	
Jmjd2c	TTGGAACGCAAATACTGGAA	CCATTCATCCACACCCTCAT	
Jmjd2b-gRNA1	CATCATTGAGGGCGTGAATAC	AGGTCCATGTCCTCTGTGTG	
Jmjd2b-gRNA2	CGCCTAGCCAATAGTGAAAAG	GACAAAGGTCAGGTTCTTCCAG	
Dynein	GACCTCAGGCTCAGACGAAGAC	AAGACGCTCATGGCATCACA	
β-Actin	ACTATTGGCAACGAGCGGTT	ACACTTCATGATGGAATTGAATGTAGT	
Cdx2	GAGAGGGCAGCGACCAGAT	CCAGGTTGGAAGGAGGAAGC	ChIP (promoter)
Eomes	CTCTGGGACCTGCCAAACTA	TTTGAAGTCTGCGAACATGG	
Gapdh	TCCTATCCTGGGAACCATCACC	TCTTTGGACCCGCCTCATTT	
Dlx2	GGCCTTTCTGGGAAACTACC	TCTGCGAAGGATGCAGAAG	ChIP (gene body)
Myf5	TTTTCAGAGGGCTGTGGTG	TGGATTGCGGTAGAAGGAAG	
Nkx2-1	ATCTGGCCAGCATGATTCAC	TTCATCTTGTAGCGGTGGTTC	
Nkx2-3	TTAAGAACCGTCGCTACAAG	CTTTACGGCCACTTTCTTGG	
Olig2	GTGGCTTCAAGTCATCTTCCTC	GGCTCAGTCATCTGCTTCTTG	
Olig3	AGATCGCCACTCTGCTTCTG	CTCTCCAACCAACCTCTTCATC	
Six1	GCTCGCCTTTGTTCACTGTC	AATTTAGGCAGGCTCTGTGG	
Sox1	TGAAGGAACACCCGGATTAC	CAGCGAGTACTTGTCCTTCTTG	
